# Multi‐Dimensional Barriers for Work Productivity and Possible Solutions to Overcome Presenteeism in Young‐Onset Diabetes: A Mixed‐Methods Study

**DOI:** 10.1111/dom.71158

**Published:** 2026-08-26

**Authors:** Kelly T. C. Wong, Jason D. C. Yin, Sunny C. S. Chan, Nga Sze Wong, Chun‐Kwan O, Yiming Dai, Yuzheng Zhang, Marques S.N. Ng, Risa Ozaki, Ronald C. W. Ma, Alice P. S. Kong, Andrea O. Y. Luk, Elaine Y.K. Chow, Juliana C. N. Chan, Juliana N. M. Lui

**Affiliations:** ^1^ Department of Medicine and Therapeutics, Faculty of Medicine The Chinese University of Hong Kong Hong Kong SAR China; ^2^ Initiative for Technology Evaluation and Cost‐effectiveness in Healthcare, Hong Kong Institute of Diabetes and Obesity The Chinese University of Hong Kong Hong Kong SAR China; ^3^ Li Ka Shing Institute of Health Science The Chinese University of Hong Kong Hong Kong SAR China; ^4^ National Center for Mental Health Beijing China; ^5^ The Nethersole School of Nursing, Faculty of Medicine The Chinese University of Hong Kong Hong Kong SAR China; ^6^ Phase 1 Clinical Trial Centre The Chinese University of Hong Kong Hong Kong SAR China; ^7^ Asia Diabetes Foundation Hong Kong SAR China

**Keywords:** distress, presenteeism, self‐care activities, work productivity, Young‐onset diabetes

## Abstract

**Aims:**

Young‐onset non‐type 1 diabetes (YOD, age‐at‐diagnosis ≤ 40) is associated with poor physical and mental health, presenteeism and productivity loss. We examined patient‐perceived barriers to work productivity and key enablers to reduce presenteeism.

**Materials and Methods:**

In this mixed‐methods study, we utilized validated questionnaires to measure presenteeism (World Health Organization Health and Performance Questionnaire); self‐care activities (Summary of Diabetes Self‐Care Activities; Compliance Questionnaire for Medication); health‐related quality of life (HRQoL, EQ‐5D‐3L) and psychological distress (Depression Anxiety Stress Scale; Chinese Diabetes Distress Scale) among participants with YOD in the Precision Medicine to Redefine Insulin Secretion and Monogenic Diabetes‐Randomised Controlled Trial (PRISM‐RCT). We conducted four semi‐structured focus group discussions (FGDs) for inductive thematic analysis.

**Results:**

Employees with YOD (*n* = 465, mean age: 43.4 ± 6.4 years, disease duration: 10.9 ± 6.0 years, 59.8% male) had better cardiometabolic, psychological and HRQoL profiles than non‐employees (*n* = 103). Among the employees, 67.4% had at least one sickness presenteeism episode in the preceding year, with a mean presenteeism score of 7.1 ± 1.6 (out of 10). Increased presenteeism was independently associated with diabetes‐related emotional burden (*β* = −0.08, *p* = 0.029) and suboptimal HRQoL (*β* = 3.30, *p* = 0.041). [Correction added on 11 September 2026, after first online publication: In the preceding sentence, the beta and p values have been corrected.] Participants of FGDs (*n* = 16) reported distress contributors related to inter‐personal (psychological/physical status, family collectivism), work (schedules/types, absence justification, colleagues' attitudes) and societal factors (relationships with providers, out‐of‐pocket payment, stigmatisation). Enablers included public awareness, early diagnosis (genetic testing for offspring, regular health‐check), peer support, treatment choices autonomy and insurance coverage.

**Conclusion:**

In YOD, presenteeism is associated with interpersonal, work and societal factors accompanied by psychological distress, suboptimal diabetes care, self‐management challenges, calling for holistic assessment and person‐centred management.

**Abbreviations**
FGD: focus group discussion
HRQoL: health‐related quality of life
YOD: Young‐onset non‐Type 1 diabetes

## 1 Introduction

The prevalence of young‐onset non‐type 1 diabetes (YOD) diagnosed before age 40 is increasing [1]. China has the largest number of people with diabetes estimated at 148 million, partly due to economic growth with shifts in demographics, environment, lifestyle and healthcare needs [2]. Patients with YOD experience accelerated decline in β‐cell function often driven by increased adiposity‐associated insulin resistance leading to premature complications and mortality [3]. These young individuals face psychological‐behavioural challenges [4] in adjusting lifestyle and adhering to the medical care plan and often experience diabetes‐related distresses due to conflicts from work and family caregiving duties [5]. Social determinants also impede annual review and attainment of target glycated hemoglobin (HbA_1c_) [6]. These multidimensional factors pertinent to personal, work, healthcare and societal factors were associated with a two‐ to sixfold increased risk of cardiovascular‐renal events and recurrent hospitalisations, notably related to mental illnesses, in YOD [7, 8, 9].

Poor quality of life, complications and impaired physical functioning co‐occurred with absenteeism (absent from work) and presenteeism (physically present at work but with reduced work performance or productivity) [10, 11]. Persistence with self‐care practice from the time of diagnosis is essential to sustain their well‐being and productivity [9, 12]. Young adulthood, particularly between the ages of 30 and 40 years, represents a critical life stage marked by career advancement and increasing family responsibilities [13]. Integrating complex self‐care routines into daily life may create diabetes distress and/or negative emotions, affecting one's daily life and their work productivity [9, 12]. Existing presenteeism‐related research focused on quantifying healthcare costs and risk factors. In the United Kingdom, psychosocial distress and impaired health‐related quality of life (HRQoL) were associated with presenteeism [14]. Sociocultural dynamics, workplace environments and health systems may also shape the experiences of people with YOD undergoing rapid socioeconomic transition [15].

In the Hong Kong Diabetes Register comprising 19 440 Chinese patients with type‐2 diabetes (T2D), onset of complications was associated with an average incremental healthcare cost of USD$2990 per patient per year, with hospitalisations accounting for the majority of the excess expenditure [16]. In an earlier report including 444 Hong Kong Chinese patients with YOD in the Precision Medicine to Redefine Insulin Secretion and Monogenic Diabetes randomised controlled trial (PRISM‐RCT), 93.9% of the participants experienced productivity loss, with a projected annual loss of USD$1.12 billion (37% from presenteeism, 63% from absenteeism). Female employees, higher health locus of control, higher salary, flexible work schedules and caregiving responsibilities for children were associated with lower presenteeism [10]. In this analysis, we further evaluated the associations of patient‐reported outcomes (PROMs) including self‐care activities, HRQoL and psychological distress with presenteeism and work productivity. We also conducted focused group discussions (FGDs) to explore possible barriers faced by people with YOD on self‐care and work that may affect productivity and enablers to overcome presenteeism loss.

## 2 Methods and Materials

### 2.1 Study Design

Between June 2023 and December 2024, we conducted a convergent‐parallel mixed‐methods study, comprising cross‐sectional analysis of factors associated with presenteeism. Nested online FGDs were conducted as an exploratory, hypothesis‐generating complement to the quantitative analysis to identify barriers, mechanisms and potential enablers of presenteeism not captured quantitatively.

### 2.2 Sample Selection and Recruitment

Participants were recruited from the PRISM‐RCT (NCT04049149) [17, 18] at the Prince of Wales Hospital, Hong Kong (Supporting Information Appendix S1). This mixed‐methods study was embedded within the ‘Economic Impact of Diabetes and Related Complications (EIDC)’ substudy of the PRISM study during Year‐3 follow‐up visits. Inclusion criteria included Chinese ethnicity, ages 18–50 with age‐at‐diabetes‐diagnosis ≤ 40 years. Patients with physician‐diagnosed type‐1 diabetes (T1D) defined as presentation with diabetic ketoacidosis and continuous insulin requirement within 12 months of diagnosis were excluded. Upon completion of the quantitative survey [10], participants who expressed interest in the follow‐up FGDs were enrolled into the recruitment pool. Participants from the pool were then purposively selected using a maximum variation sampling strategy to ensure heterogeneity of perspectives, guided by four pre‐defined diversity criteria: *employment types*, *work schedules*, *presence of complications* and *use of insulin*.

### 2.3 Measures and Procedures

#### 2.3.1 Study Assessments

Presenteeism was measured by the World Health Organization Health and Performance Questionnaire (WHO‐HPQ) [19] using a self‐assessed item ‘How would you rate your overall job performance on the days you worked during the past 4 weeks (28 days)?’ expressed on a 10‐point Likert scale (0—worst performance; 10—top performance), with lower scores indicating greater presenteeism (i.e., productivity loss). A cut‐off score of ≤ 40 (after multiplying the presenteeism score by 10) was used to indicate high‐presenteeism [20].

We used the following PROMs to measure psychological distress, HRQoL and self‐care activities: Depression Anxiety Stress Scales (DASS‐21) [21]; EuroQoL 5‐Dimension 3‐Level (EQ‐5D‐3L) [22, 23]; Chinese version of the Diabetes Distress Scale (CDDS‐15) [24]; Summary of Diabetes Self‐Care Activities (SDSCA‐15) [25] and Compliance Questionnaire (CQ‐4) [26] along with documentation of demographic and socioeconomic characteristics, medical history and medication use. Cardiometabolic risk factors included fasting plasma glucose, HbA_1c_, high‐density lipoprotein cholesterol (HDL‐C), low‐density lipoprotein cholesterol (LDL‐C), triglycerides (TG), systolic (SBP) and diastolic blood pressure (DBP) and markers of kidney function (spot urine albumin‐to‐creatinine ratio [uACR] and estimated glomerular filtration rate[eGFR]) [27].

#### 2.3.2 Qualitative Semi‐Structured FGD Study Design and Procedures

We designed a question guide covering decision‐making processes underlying presenteeism, the influence of workplace and human‐resource policies on employees' ability to manage their health and the socioeconomic impact of diabetes and its complications on employees living with the condition (Supporting Information Appendix S2). We conducted the semi‐structured FGD by Zoom meeting. Each FGD comprised three to five participants and lasted 60–90 min, conducted sequentially by the lead researcher and a trained research assistant. Chinese verbatim transcripts were preliminarily coded after each session. Data collection ceased upon unanimous agreement on thematic saturation when no new codes emerged from subsequent FGDs. Saturation was achieved after four FGDs with themes redundancy which fell within a recommended range of 4–8 FGDs [28].

### 2.4 Statistical Analysis

Data were summarised as mean ± standard deviation (SD), median (interquartile range, IQR) or percentages. Group comparisons were performed using independent *t*‐tests and Mann–Whitney *U* tests, as appropriate. We used hierarchical linear regression, adjusted for covariates, to evaluate associations of psychological health, HRQoL and self‐care activities with presenteeism. Model‐1 included demographics; Model‐2 additionally adjusted for cardiometabolic risk factors and Model‐3 further added medications (Supporting Information Appendix S1).

Thematic analysis was conducted using NVivo14. Four researchers inductively coded transcripts following Braun and Clarke's six‐step framework (Supporting Information Appendix S1), allowing codes to emerge directly from the data [29]. The researchers' gender, roles, qualifications and reflexivity are described in Supporting Information Appendix S1. The team met after the interview to compare, discuss and refine codes to reach consensus. Emergent themes were organised into a multi‐level framework derived inductively from the data rather than defined a priori. Joint display diagrams were constructed and meta‐inferences were generated to integrate the quantitative and qualitative findings.

**TABLE 1:** Mixed‐methods joint display of findings and meta‐inferences.

Quantitative findings^a^	Qualitative findings		Meta‐inferences
Comparisons	Contributing factors	Healthcare enablers	
↑ Presenteeism group had …			
↑ Stress	Psychological and physical strain	Peer support group	The high‐presenteeism group experienced higher overall stress, which may come from work‐life imbalance, self‐care demands and family responsibilities. Peer support might alleviate loneliness and isolation while strengthen their capacity for diabetes management.
↓ Dietary adherence	Cultural collectivism Psychological and physical strain Work types and schedules Concerns about the attitude of employers and colleagues	Healthy lifestyle promotion Peer support group	Participant perceived psychological and physical strain could trigger emotional eating; while family and workplace environments or schedules could lead to irregular meal times and limited food choices. Healthy lifestyle promotion not only expands food choices, but also equips individuals with healthier dietary adherence.
↑ Working hours; ↑ sickness attendance	Concerns about the attitude of employers and colleagues Societal perception	Genetic screening and regular body check Educational programme	Long working hours and sickness attendance were prominent among the high‐presenteeism group. This pattern is likely driven by fears of workplace stigmatisation (e.g., diabetes signals incompetence), compounded by widespread misunderstandings of the disease. Educational programmes and genetic screening/regular health checks could raise public awareness, correct misconceptions and enhance self‐management.
↑ Regimen‐and‐social‐support‐related distress	Medical costs Relationships with care providers	Patient involvement in shared decision‐making Conditional insurance coverage Genetic screening and regular body check	The regimen‐ and social support‐related distress may arise from effective yet high‐cost treatment, lack of autonomy treatment planning and insufficient trust in providers. Involving patients in shared decision‐making and offering genetic screening may empower them over their condition and treatment; while conditional insurance could help ease their financial burden, thus, reduce their distress.
Associations			
↑ Diabetes‐related emotional burden predicted ↑ presenteeism	Challenges in applying for leaves Work types and schedules	Peer support group Healthy lifestyle promotion Educational programme	Participants reported significant challenges in balancing work and self‐care. Inflexible company policies and demanding work schedules were cited as key barriers, resulting in missed medical follow‐ups and increased presenteeism. Some participants changed their job to better manage their health. Peer support, healthy lifestyle promotion and educational programmes could raise awareness on health priorities, reduce emotional burden and improve HRQoL, thereby lowering presenteeism.
↓ HRQoL predicted ↑ presenteeism	Psychological and physical strain Medical costs Work types and schedules		

^a^↑ = higher level, ↓ = lower level.

## 3 Results

### 3.1 Quantitative Findings

In this EIDC substudy, among 568 Chinese participants with YOD, 82% (*n* = 465) were employees (Figure 1). Employees were more likely to be men, older at diagnosis, carers of child(ren) under 18‐year‐old and had better cardiometabolic risk factors and lower prevalence of all‐site cancer than non‐employees (Table S1). Among the employees, the weekly working hours were 48.9 ± 14.0. One‐third of them earned HKD18 700–29 499/month (USD$1 = HKD$7.8). 67.4% reported at least one episode of sickness presenteeism last year, with a mean presenteeism score (i.e., productivity loss severity) of 7.1 ± 1.6 (out of 10) (Table S1). Overall, one in three had diabetes‐related distress (CDSS‐15 ≥ 45).

**FIGURE 1:**
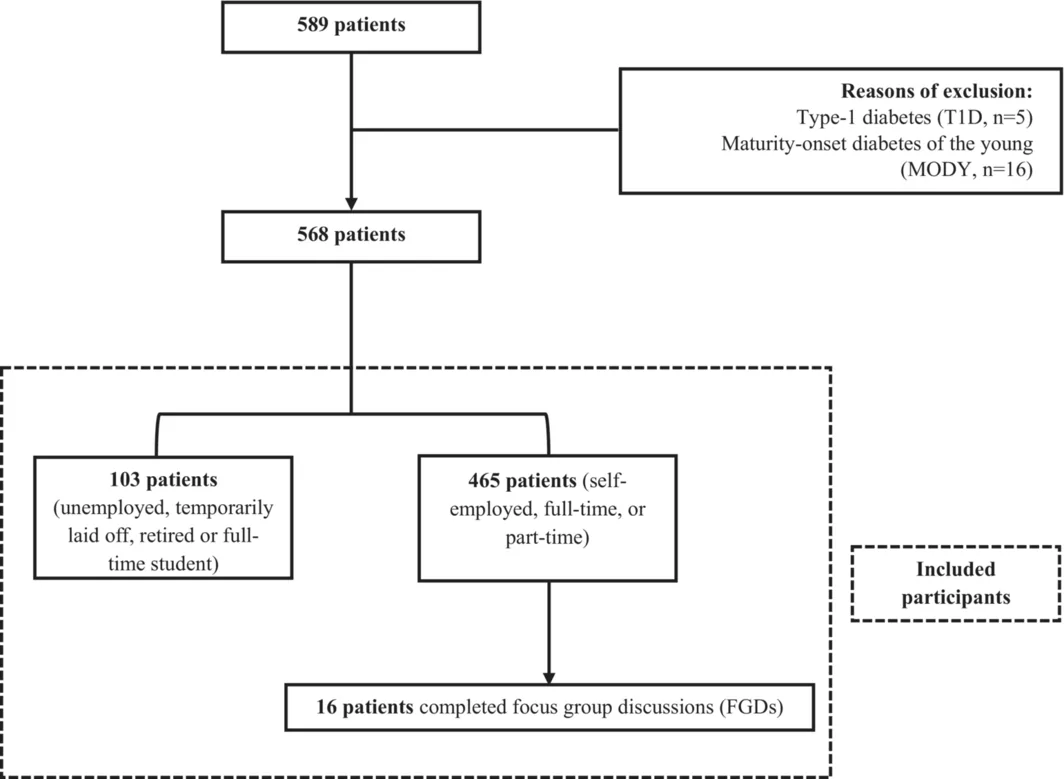
CONSORT diagram of subjects included in the analysis.

Among these participants, 23 (5.0%) reported presenteeism score ≤ 40 (range 0–100). The high‐presenteeism group reported longer working hours of 55.4 ± 14.8 per week, a higher rate of sickness attendance in the past year (91.3%), poorer HRQoL, higher stress, higher regimen‐and‐social‐support‐related distress and poorer dietary adherence than the low‐presenteeism group (Table 2).

**TABLE 2:** Demographic characteristics of employed participants in the substudy of the PRISM‐RCT stratified by high‐ versus low‐presenteeism.

	Frequency (%)/mean ± SD/median (interquartile range)				*p*
	*N*	Low‐presenteeism (*N* = 438)	*N*	High‐presenteeism (*N* = 23)	
Demographic characteristics					
Age (years)	438	43.4 ± 6.4	23	42.9 ± 6.4	0.678
Men (*n*, %)	438	259 (59.1)	23	17 (73.9)	0.233
College or above (*n*, %)	438	170 (38.8)	23	10 (43.5)	0.820
Taking care of children < 18‐year (*n*, %)	438	145 (33.1)	23	9 (39.1)	0.711
Current or former tobacco use (*n*, %)	438	143 (32.6)	23	8 (34.8)	1.000
Never drank alcohol (*n*, %)	438	254 (58.0)	23	10 (43.5)	0.248
Family history of diabetes (*n*, %)	432	344 (79.6)	22	19 (86.4)	0.619
Metabolic risk factors					
Age at diagnosis (years)	438	33.0 ± 6.4	23	33.0 ± 6.4	0.993
Duration of diabetes (years)	438	10.9 ± 6.0	23	10.4 ± 5.7	0.706
Fasting plasma glucose (mmol/L)	436	7.4 ± 2.4	23	7.4 ± 1.6	0.992
HbA_1c_ (%)	436	7.3 ± 1.5	23	7.3 ± 1.0	0.983
HbA_1c_ < 7% (*n*, %)	436	208 (47.7)	23	14 (60.9)	0.309
Body mass index (kg/m^2^)	435	28.1 ± 5.3	23	28.0 ± 3.6	0.953
Waist circumference (men, cm)	203	100.0 ± 12.4	12	96.8 ± 7.4	0.390
Waist circumference (women, cm)	146	93.5 ± 12.9	5	103.2 ± 6.9	0.094
Systolic BP (mmHg)	435	125.6 ± 15.1	23	121.9 ± 13.2	0.241
Diastolic BP (mmHg)	435	75.9 ± 10.3	23	74.1 ± 11.5	0.420
LDL‐cholesterol (mmol/L)	426	2.1 ± 0.8	23	2.0 ± 0.9	0.663
Triglycerides (mmol/L)	436	1.3 (0.9, 2.0)	23	1.6 (1.0, 2.0)	0.460
eGFR (mL/min/1.73 m^2^)	436	104.6 (89.1, 111.3)	22	104.7 (93.7, 110.2)	0.953
Albuminuria (*n*, %)	438	148 (33.8)	23	9 (39.1)	0.763
Diabetes self‐management and hypoglycaemia					
Self‐reported hypoglycaemia in last 3 months (*n*, %)	438	109 (24.9)	23	3 (13.0)	0.298
Self‐monitoring of blood glucose/week (*n*, %)	438	146 (33.3)	23	7 (30.4)	0.952
Physical activity ≥ 5 times/week (*n*, %)	438	84 (23.9)	23	4 (25.0)	1.000
Good adherence to balanced diet (*n*, %)	438	98 (22.4)	23	2 (8.7)	0.196
Medication					
Oral glucose‐lowering drugs (*n*, %)	438	412 (94.1)	23	22 (95.7)	1.000
Insulin (*n*, %)	438	124 (28.3)	23	3 (13.0)	0.174
GLP‐1 RA (*n*, %)	433	113 (26.1)	21	5 (23.8)	1.000
Blood pressure‐lowering drugs (*n*, %)	438	343 (78.3)	23	19 (82.6)	0.819
Renin angiotensin system inhibitor (*n*, %)	430	310 (72.1)	21	15 (71.4)	1.000
Lipid‐regulating drugs (*n*, %)	438	360 (82.2)	23	22 (95.7)	0.166
Statin (*n*, %)	430	344 (80.0)	20	17 (85.0)	0.794
History of complications and comorbidities					
Cardiovascular disease (*n*, %)	438	44 (10.0)	23	0 (0.0)	0.217
Coronary heart disease (*n*, %)	438	29 (6.6)	23	0 (0.0)	0.404
Stroke (*n*, %)	438	11 (2.5)	23	0 (0.0)	0.945
Peripheral vascular disease (*n*, %)	438	6 (1.4)	23	0 (0.0)	1.000
Congestive heart failure (*n*, %)	438	2 (0.5)	23	0 (0.0)	1.000
Chronic kidney disease (*n*, %)	438	31 (7.1)	23	1 (4.3)	0.935
End‐stage kidney disease (*n*, %)	438	4 (0.9)	23	1 (4.3)	0.605
All‐site cancer (*n*, %)	438	15 (3.4)	23	1 (4.3)	1.000
Presenteeism					
Working hours per week (hours)	435	48.6 ± 13.7	23	55.4 ± 14.8	**0.023**
Presenteeism score (0–10)^a^	438	7.3 ± 1.3	23	3.4 ± 0.9	**< 0.001**
Patient‐reported outcomes					
EQ‐5D‐3L utility score	429	0.9 ± 0.1	23	0.8 ± 0.2	**0.001**
EQ‐5D‐3L VAS (0–100)	433	69.3 ± 16.8	23	64.0 ± 13.2	0.136
DASS‐21					
Depression (0–21)	432	1.5 (0.0, 5.0)	23	2.0 (1.5, 6.0)	0.056
Anxiety (0–21)	433	2.0 (1.0, 5.0)	23	3.0 (2.0, 5.5)	0.058
Stress (0–21)	433	4.0 (0.0, 8.0)	23	6.0 (2.0, 8.5)	**0.033**
C‐DDS‐15					
Emotional burden (6–36)	431	15.9 ± 6.5	23	18.1 ± 6.4	0.112
Regimen‐ and social‐related distress (6–36)	431	16.8 ± 5.8	23	19.6 ± 5.7	**0.025**
Physical‐related distress (3–18)	430	5.8 ± 2.7	23	5.3 ± 2.1	0.399
C‐DDS‐15 ≥ 45	428	125 (29.2)	23	9 (39.1)	0.435
SDSCA‐15					
Diet (0–7)	433	3.5 (2.8, 4.5)	23	3.0 (2.0, 3.5)	**0.004**
Exercise (0–7)	433	2.0 (1.0, 3.5)	23	1.5 (0.5, 2.5)	0.101
Blood sugar testing (0–7)	431	1.0 (0.0, 3.0)	23	1.0 (0.0, 2.0)	0.818
Foot care (0–7)	433	3.3 (2.3, 5.0)	23	3.0 (2.3, 3.8)	0.116
Adherence to medical advice (0–7)	174	4.7 (2.3, 6.3)	6	3.8 (2.8, 4.7)	0.511
CQ‐4 Total (0–4)	427	4.0 (3.0, 4.0)	23	3.0 (3.0, 4.0)	0.165

*Note: p*‐values less than 0.05 were shown in bold.

Abbreviations: C‐DDS‐15, Chinese version of Diabetes Distress Scale—15 items; CQ‐4, Compliance Questionnaire—4 items; DASS‐21, Depression, Anxiety and Stress Scales—21 items; EQ‐5D‐3L, EuroQol 5 Dimension 3 Level Version; SDSCA‐15, Summary of Diabetes Self‐Care Activities—15 items; VAS, Visual Analogue Scale.

*p*‐values indicate the comparison between the high‐ and low‐presenteeism groups.

In the multivariate analysis including all PROMs, higher HRQoL and lower emotional distress (CDDS‐15) were independently associated with lower presenteeism. These associations remained significant after adjusting for demographics, cardiometabolic risks and medications (Table 3). Consistent findings were observed in separate models examining each PROM in relation to presenteeism (Table S2).

**TABLE 3:** Multivariable linear regression analysis of the associations of presenteeism with psychological health and self‐care activities.

	Model‐1					Model‐2					Model‐3				
	*β*	SE	95% CI	VIF	*p*	*β*	SE	95% CI	VIF	*p*	*β*	SE	95% CI	VIF	*p*
DASS‐21															
Depression	−0.06	0.07	−0.19, 0.08	5.82	0.392	−0.08	0.08	−0.23, 0.07	6.55	0.288	−0.05	0.08	−0.21, 0.10	6.10	0.501
Anxiety	0.01	0.06	−0.11, 0.13	3.55	0.893	0.05	0.07	−0.09, 0.18	4.24	0.507	0.06	0.07	−0.08, 0.20	4.08	0.393
Stress	0.06	0.05	−0.04, 0.16	4.71	0.276	0.06	0.05	−0.05, 0.17	5.06	0.264	0.03	0.06	−0.08, 0.15	5.54	0.578
C‐DDS‐15															
Emotional burden	−0.06	0.03	−0.12, 0.00	3.12	0.056	−0.08	0.03	−0.14, −0.01	3.41	**0.019**	−0.08	0.04	−0.15, −0.01	3.83	**0.029**
Regimen‐and‐social‐related distress	0.04	0.03	−0.03, 0.10	2.84	0.298	0.04	0.04	−0.04, 0.11	3.04	0.334	0.05	0.04	−0.03, 0.13	3.36	0.190
Physician‐related distress	0.05	0.05	−0.05, 0.14	1.43	0.330	0.04	0.05	−0.06, 0.13	1.39	0.480	0.05	0.05	−0.05, 0.15	1.38	0.339
EQ‐5D‐3L utility score	3.54	1.27	1.03, 6.04	2.36	**0.006**	3.66	1.34	1.02, 6.31	2.49	**0.007**	3.30	1.60	0.14, 6.46	2.61	**0.041**
SDSCA															
Diet	0.11	0.11	−0.12, 0.33	1.75	0.342	0.12	0.12	−0.12, 0.36	1.92	0.331	0.18	0.13	−0.08, 0.44	2.15	0.177
Exercises	−0.04	0.08	−0.20, 0.11	1.38	0.560	−0.08	0.08	−0.24, 0.09	1.53	0.351	−0.11	0.09	−0.28, 0.06	1.64	0.188
Blood sugar testing	−0.08	0.07	−0.22, 0.06	1.50	0.256	−0.11	0.08	−0.26, 0.04	1.58	0.144	−0.13	0.08	−0.29, 0.03	1.79	0.111
Foot care	0.22	0.08	0.07, 0.37	1.41	**0.004**	0.21	0.08	0.04, 0.37	1.62	**0.013**	0.16	0.09	−0.01, 0.34	1.79	0.063
Medication adherence	0.04	0.07	−0.09, 0.17	1.49	0.574	0.01	0.07	−0.13, 0.16	1.70	0.851	0.03	0.09	−0.14, 0.21	2.29	0.713
CQ‐4	−0.02	0.11	−0.25, 0.20	1.53	0.836	−0.06	0.13	−0.31, 0.19	1.85	0.632	0.00	0.14	−0.27, 0.27	2.03	0.998

*Note:* Presenteeism, “How would you rate your overall job performance on the days you worked during the past 4 weeks (28 days)?” from World Health Organization‐Health and Work Performance Questionnaire (WHO‐HPQ); Model‐1: Demographics (age, sex, duration of diabetes, college or above, taking care of child, group assignment); Model‐2: Model‐1 + Cardiometabolic risk factors (BMI, HDL‐C, LDL‐C, SBP, DBP, TG, eGFR, HbA1c, fasting plasma glucose); Model‐3: Model‐2 + Medications (antidiabetic drugs, insulin, GLP‐1, antihypertensive drugs, RASi, hyperlipidaemia agents, statin) were adjusted. Regression was conducted with all the PROMs (i.e., DASS‐21, C‐DDS‐15, EQ‐5D‐3L utility score, SDSCA and CQ‐4) entered to the model simultaneously. *p*‐values indicate the association between study variables and presenteeism, *p*‐values less than 0.05 were shown in bold.

Abbreviations: C‐DDS‐15, Chinese version of Diabetes Distress Scale—15 items; CQ‐4, Compliance Questionnaire—4 items; DASS‐21, Depression, Anxiety and Stress Scales—21 items; EQ‐5D‐3L, EuroQol 5 Dimension 3 Level Version; SDSCA‐15, Summary of Diabetes Self‐Care Activities—15 items; VIF, variance inflation factor.

### 3.2 Qualitative Findings

Of the 465 employees invited, 115 (24.7%) agreed to participate in FGDs, of whom 16 were purposively selected and assigned to four FGDs (mean age: 45.1 ± 5.9 years, age‐at‐diagnosis: 33.6 ± 6.8 years, diabetes duration: 2.1 ± 7.1 years). The clinical characteristics of FGD participants and non‐participants had comparable working profiles and presenteeism score (7.1 ± 1.6 vs. 7.1 ± 1.5 respectively) (Table S3). We performed comparative analysis between the employed and unemployed, high‐ and low‐presenteeism and FGD and non‐FGD groups using *p*‐values with false discovery rate (FDR) adjusted for multiple comparisons (Table S4).

The qualitative analysis generated 15 codes, which were organised into two central categories: *contributing factors* and *healthcare policies*, each with three sub‐themes. Thematic prevalence table and example quotes under each subtheme were included in Tables 4 and S5, respectively.

#### 3.2.1 Contributing Factors

The *contributing factors* included *personal factors*, *workplace environment* and *societal influences* (Figure 2a).

**FIGURE 2:**
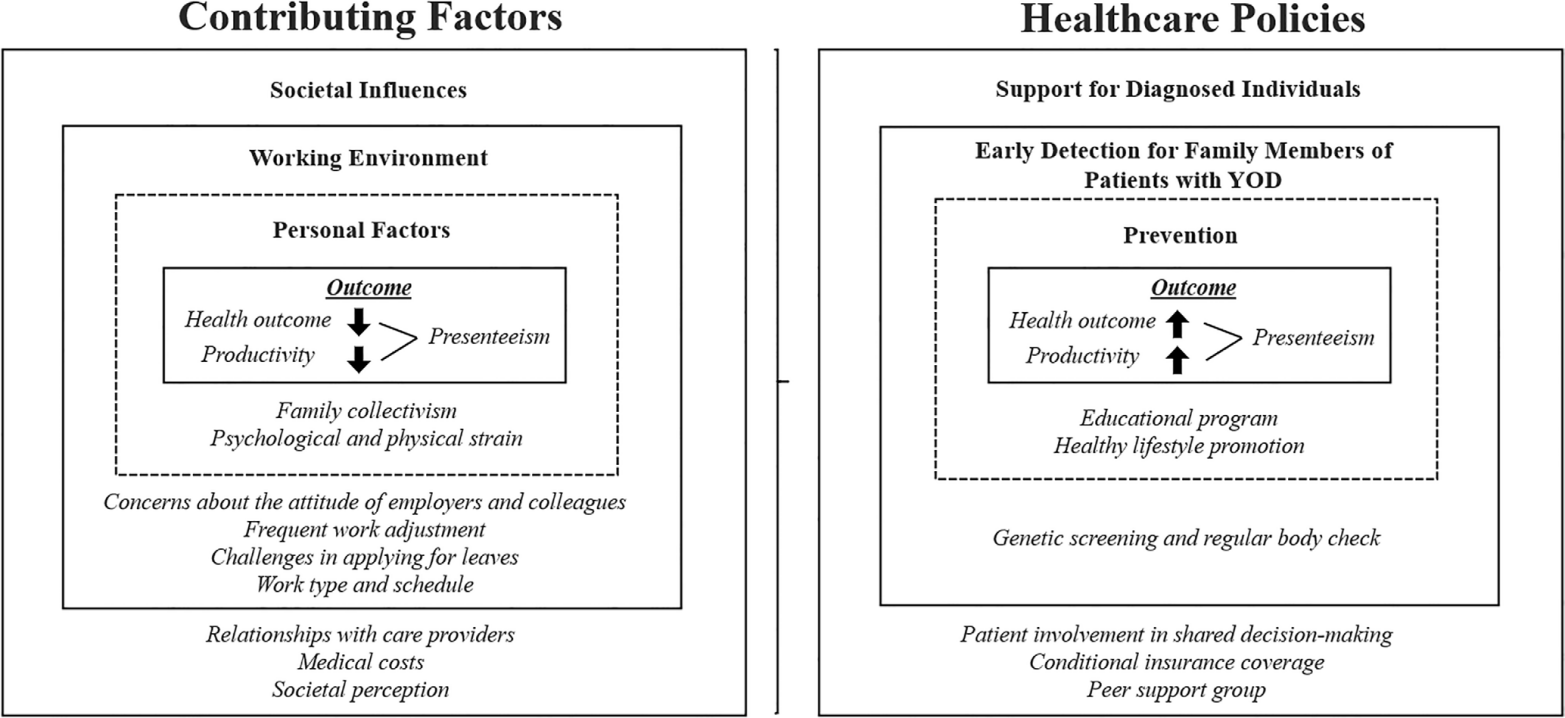
Multi‐level framework on employee outcomes of presenteeism, productivity and diabetes management based on (a) contributing factors and (b) healthcare policies.

**TABLE 4:** Prevalence of themes identified from focus group discussions.

Theme	Number of participants (*n* = 16) Frequency (%)	Prevalence category^a^
Contributing factors		
Personal factors		
Cultural collectivism	5 (31.3)	Common
Psychological and physical strain	7 (43.8)	Common
Working environment		
Concerns about the attitude of employers and colleagues	11 (68.8)	Majority
Frequent work adjustment	9 (56.3)	Common
Challenges in applying for leave	11 (68.8)	Majority
Work types and schedules	8 (50.0)	Common
Societal influences		
Relationships with care providers	7 (43.8)	Common
Medical costs	10 (62.5)	Majority
Societal perception	3 (18.8)	Minority
Healthcare policies		
Prevention		
Educational programme	7 (43.8)	Common
Healthy lifestyle promotion	3 (18.8)	Minority
Early detection for family members of patients with YOD		
Genetic screening and regular body check	4 (25.0)	Minority
Ongoing support		
Patient involvement in shared decision‐making	3 (18.8)	Minority
Conditional insurance coverage	8 (50.0)	Common
Peer support group	3 (18.8)	Minority

^a^ Majority theme = ≥ 60% of participants; Common theme = 35%–59%; minority theme = < 35%.

##### 3.2.1.1 Personal Factors

###### 3.2.1.1.1 Family Collectivism

Commensality and shared meals are norms in many Asian families. Waiting for other family members to return from work often delayed meals. Sharing meals with children and older relatives without chronic medical conditions posed barriers to healthy food choices. Desire to encourage a nutrient‐balanced diet within the households could be met by resistance from other family members. Caregiving responsibilities could increase stress and fatigue, which in turn impeded effective health management (respondent‐10‐FGD‐2). Family reciprocity and support, especially spouses with similar medical conditions, reinforced adherence to self‐monitoring and medication (respondent‐14‐FGD‐4).

###### 3.2.1.1.2 Psychological and Physical Strain

Work‐related stress contributed to binge eating or increased food cravings with high content of sugar or sodium glutamate (respondent‐15‐FGD‐4; respondent‐16‐FGD‐4). In Hong Kong, eating is a convenient way to relieve stress (respondent‐11‐FGD‐3), posing challenges in adhering to a balanced diet despite having the appropriate knowledge (respondent‐4‐FGD‐1).

##### 3.2.1.2 Working Environment

###### 3.2.1.2.1 Concerns About the Attitude of Employers and Colleagues

Colleagues were perceived to view diabetes as a reflection of ineffective self‐management and vulnerability. The was in contrast to greater societal acceptance for hypertension with more stigmatisation for diabetes, obesity and associated treatments (e.g., injections) (respondent‐14‐FGD‐2). Disclosure of diabetes diagnosis might negatively influence employers' and colleagues' perceptions of competence, performance evaluation and career advancement (respondent‐4‐FGD‐1; respondent‐7‐FGD‐2). Participants experienced ambivalence when faced with decision of declining meal invitations or refusing certain foods during significant team events (respondent‐6‐FGD‐2). Supporting workplace cultures (respondent‐16‐FGD‐4) and health‐literate colleagues facilitated acceptance, often with friendly reminders on food choices/portions and health tips (respondent‐4‐FGD‐1).

###### 3.2.1.2.2 Frequent Work Adjustment

Regular medical visits (e.g., half‐day hospital visits every 3 months), biennial complication assessments and education sessions disrupted work schedule requiring temporary staff substitution (respondent‐4‐FGD‐1). These challenges sometimes prompted the acceptance of a lower‐intensity position with reduced earnings (respondent‐7‐FGD‐2).

###### 3.2.1.2.3 Challenges in Applying for Leave

Some supervisors only accepted sick leave certificates issued by a doctor, posing challenges for assessments or education by non‐medical staff, who could only issue certificates of attendance in Hong Kong public healthcare settings (respondent‐5‐FGD‐1).

###### 3.2.1.2.4 Work Types and Schedules

Shift‐work hindered adherence to a healthy lifestyle due to irregular mealtimes (respondent‐15‐FGD‐4) and late working hours. These non‐routine working hours reduced frequencies of home cooking with frequent dining out with unbalanced diets (respondent‐1‐FGD‐1; respondent‐9‐FGD‐2). These challenges were often underappreciated by clinicians (respondent‐1‐FGD‐1).

##### 3.2.1.3 Societal Influences

###### 3.2.1.3.1 Relationships With Care Providers

Many respondents were knowledgeable about advanced diabetes technologies, such as continuous glucose monitoring (CGM) devices and used them to support self‐management (respondent‐9‐FGD‐2). These efforts were not fully recognised by doctors who preferred using medications to achieve control (respondent‐4‐FGD‐1). Limited personalisation, brief consultations and fragmented care in public settings reduced perceived usefulness of advice, contributing to disengagement and mistrust (respondent‐14‐FGD‐4, respondent‐6‐FGD‐2).

###### 3.2.1.3.2 Medical Costs

High costs of innovative medications (e.g., GLP1‐RA) and technologies (e.g., CGM) were major barriers. In Hong Kong. In public hospitals/clinics, coverage for GLP1‐RA subsidies was restricted to patients with obesity who had complications or multiple risk factors. Many young patients with overweight but without complications had to pay out of pocket with frequent discontinuation (respondent‐6‐FGD‐2, respondent‐14‐FGD‐4). Lack of or insufficient insurance coverage for diabetes‐related expenses, disqualification due to the ‘inherited’ nature of diabetes (respondent‐5‐FGD‐1) and additional premium loadings (respondent‐4‐FGD‐1) further constrained access.

###### 3.2.1.3.3 Societal Perception

Limited health literacy of the public on YOD, misconceptions that diabetes is a disease of the elderly and that young age is a disease‐free state hindered discussion between individuals with YOD and those without diabetes (respondent‐15‐FGD‐4).

#### 3.2.2 Healthcare Policies

Several potential enablers proposed by the participants were listed in the *healthcare policies* category, including *prevention*, *early detection* and *personalised treatment* (Figure 2b).

##### 3.2.2.1 Prevention

###### 3.2.2.1.1 Educational Programme

Preventive efforts should incorporate early public education on diabetes, targeting children and their parents to increase awareness and promote healthy behaviours from a young age. Curricula should include explanation on diabetes subtypes, healthy lifestyle practices, correction of misconceptions that diabetes is a ‘geriatric disease’ and de‐stigmatisation of diabetes‐related practices (e.g., injections, CGM, self‐monitoring of blood glucose) (respondent‐11‐FGD‐3). Early life education that promotes regular meals, physical activity and healthy nutrition (respondent‐16‐FGD‐4) was viewed as a long‐term strategy to improve individual and societal well‐being (respondent‐3‐FGD‐1).

###### 3.2.2.1.2 Healthy Lifestyle Promotion

Other suggestions included government policies aimed at encouraging restaurants and caterers to offer healthier meal choices, especially for individuals with limited cooking time (respondent‐12‐FGD‐3).

##### 3.2.2.2 Early Detection for Family Members of Patients With YOD

###### 3.2.2.2.1 Genetic Screening and Regular Body Check

Genetic screening and regular health check‐ups for complications were suggested to enable early detection, raise awareness and motivate healthier lifestyles (respondent‐11‐FGD‐3). Addressing concerns about inherited risk and improving access to genetic testing with clear action plans for offspring to mitigate risk were highlighted (respondent‐13‐FGD‐3).

##### 3.2.2.3 Ongoing Support

###### 3.2.2.3.1 Patient Involvement in Shared Decision‐Making

Insufficient explanation by physicians regarding medication options, benefits and risks hindered informed decision‐making. Clearer personalised information could increase willingness to pay out‐of‐pocket for organ‐protective therapies as a long‐term health investment (respondent‐11‐FGD‐3; respondent‐12‐FGD‐3).

###### 3.2.2.3.2 Conditional Insurance Coverage

Conditional insurance incentives for sustained glycaemic control (HbA_1c_ < 7%) were suggested to encourage self‐care and reduce financial burden (respondent‐8‐FGD‐2).

###### 3.2.2.3.3 Peer Support Group

Feelings of loneliness and isolation might be relieved through peer knowledge‐sharing and learning on self‐care (respondent‐7‐FGD‐2), preferably via online platforms or chat groups that do not require immediate response due to different work schedules (respondent‐9‐FGD‐2).

### 3.3 Mixed‐Methods Integration

Table 1 presents a joint display diagram with meta‐inferences. Quantitative analysis on diabetes‐related emotional burden and HRQoL with presenteeism converged with qualitative themes. The latter included challenges in submitting leave applications, psychological and physical strain, financial burden of medical care and variation in work types and schedules. Inflexible company policies and demanding schedules were identified as barriers to attending medical follow‐ups and contributors to work–health conflicts.

Concerns over employers' and colleagues' attitudes, alongside societal perceptions, influenced decisions related to presenteeism. Financial strain on medical care and patient‐provider relationships underlie the heightened regimen‐and‐social support‐related distress among participants with high presenteeism. Cultural norms emphasising collectivism also appeared to influence self‐care practices.

## 4 Discussion

In this study, we applied a convergent parallel mixed‐methods design, utilising validated questionnaires to explore the associations of psychological distress, HRQoL and self‐care activities with presenteeism. This was complemented by semi‐structured interviews to elucidate underlying barriers and enablers.

### 4.1 Vicious Cycle of Work, Stress and Collectivism Challenging Self‐Care Adherence

Work stress and caregiving responsibilities were weighted heavily by our employed interviewees. Both high‐ and low‐presenteeism groups had long working hours (48–55 h/week). In Hong Kong, these long working hours had been associated with cardiovascular diseases [30]. Consistent with prior evidence, working hours ≥ 48/week were accompanied by frequent fatigue and exhaustion, especially for those with childcare responsibilities. Some participants reported binge eating as a stress‐coping strategy, a behaviour linked to obesity [31] and depression [32, 33].

In China, the prevalence of overweight/obesity had risen from 3.1% in 2004 to 16.4% in 2023 [34]. Our qualitative findings suggest a potential sequential pathway comprising work‐related stress precipitating emotional eating with consequent weight gain, deterioration in glycaemic control and heightened presenteeism. Other researchers had reported that obesity and stress‐related eating behaviours were associated with nearly a twofold increase in workplace presenteeism. Excess adiposity may exacerbate insulin resistance and impair β‐cell function [35] with ineffective energy metabolism which may diminish work capacity and performance [36]. This stress–eating–weight cycle warrants rigorous investigation, as it may perpetuate a self‐reinforcing pattern of weight gain, psychological distress, suboptimal health and increased presenteeism.

Work‐health conflicts arose when occupational demands impeded diabetes self‐management [37]. Within Chinese collectivist culture, shared meals are central to family and workplace relationships [38, 39]. Finishing a meal is etiquette, while offering food expresses hospitality [40]. Consistent with prior reports [41], our participants reported pressure and embarrassment with food choices as a major barrier to dietary adherence. Work schedules also exerted complex effects. Shift work may disrupt circadian rhythms [42] although in our prior analysis, this was linked with lower presenteeism, possibly reflecting greater flexibility and reduced sick‐leave‐related stress [10]. Participants also cited Hong Kong‐specific administrative barriers where non‐acceptance of sick‐leave certificates issued by non‐physician care providers might limit access to multidisciplinary care. These constraints may coincide with frequent defaults for educational programmes and suboptimal diabetes control [37]. These practical barriers highlight the sociocultural work‐health conflicts and system‐level gaps calling for flexible policies, such as acceptance of leave for educational or assessment sessions in addition to medical consultations.

Diabetes‐related emotional distress and HRQoL remained significantly associated with presenteeism after covariate adjustment. These findings suggest that diabetes‐specific psychological factors may affect work performance, independent of physical illness burden and mechanistically, may be mediated by a pathway distinct from cardiometabolic indices. In keeping with the negative emotions associated with pill burden, depressive symptoms were attenuated after adjustment for medication use [43]. Despite the high prevalence of stress among those with high presenteeism, there was no clear relationship between stress and depression. These dissociative patterns may reflect differences in how condition‐specific versus general mood constructs may have different effects on work functioning.

Overall, work‐ and diabetes‐specific demands may represent more proximal influences on presenteeism than general mood symptoms in employees with YOD. This interpretation aligns with the notion that the day‐to‐day burden of diabetes management, including sustained behavioural adaptation, self‐monitoring and treatment adherence, may exert more immediate effects on occupational performance than generalised psychological distress.

Our participants identified peer support as a potentially valuable strategy. Peer‐based interventions have been associated with improvements in cardiometabolic outcomes, self‐care behaviours, emotional well‐being and healthcare utilisation [44, 45, 46]. Future research should evaluate such interventions in our workforce with diabetes using larger, representative samples with real‐time assessment and prospective follow up for clinical outcomes. This holistic approach incorporating biomedical‐cognitive‐psychological‐behavioural intervention will better characterise the temporal and causal relationships among psychological burden, work functioning, clinical outcomes and societal productivity.

### 4.2 Health Literacy and Diabetes Stigmatisation in Presenteeism

Several participants noted a lay misconception that diabetes exclusively affects older individuals. Stigma thrives in power‐laden social contexts [47, 48], such as workplace, where hierarchies, scrutiny and professional consequences converge. Diabetes‐related attributes, notably obesity and visible self‐care activities (e.g., fingerpricks, insulin injections), are often misattributed to personal failings [48, 49]. These negative perceptions foster ‘ingroup’ versus ‘outgroup’ comparisons, creating automatic stereotype that culminate status loss and discrimination [47, 48]. Experiences of *felt stigma* (i.e., shame and anticipation of judgement) [50] coincided with presenteeism as reported by the participants. These included concealment of their condition, avoidance of taking sick leave for fear of jeopardising job security and embarrassment when declining food at social occasions. These complex internal and external experiences can adversely affect physical and mental health as well as productivity, with particular relevance to YOD [51]. Similarly, low public awareness of diabetes risk factors, subtypes, pathophysiology and treatment has been documented in a Singapore study [52]. Enhancing public awareness that diabetes can affect individuals of any age, body weight or ethnicity may support prevention and reduce stigma, as echoed by our participants.

### 4.3 Care Gaps, Patient Autonomy, Trust and Financial Barriers

High regimen‐and‐social‐support‐related distress was observed in the high‐presenteeism group with insufficient patient‐provider engagement and variations in health knowledge. Consistent with prior findings [53], some respondents recognised the potential of diabetes technologies to improve health behaviours but perceived insufficient clinical support to utilise them effectively. This misalignment may undermine patient‐provider engagement and trust. Genetic testing was raised during focus group discussions. Some participants suggested that such information could enhance health awareness and support preventive efforts. In the PRISM‐RCT study where we recruited this sub‐cohort, genetic testing was associated with improvements in metabolic control, β‐cell function indices and patient‐provider communications [17]. Shifting from paternalistic doctor–patient relationships towards promoting health literacy and patient autonomy could enhance HRQoL and positive behaviour changes [54] which may reduce presenteeism. The growing trend towards a collaborative model [55] demands healthcare system reform to empower patients' decision‐making. This approach is particularly relevant to YOD, where complex and fragmented care prevail. These achievable factors, perceived disease autonomy and empowerment, may mitigate presenteeism.

Financial barriers can lead to suboptimal care with direct and indirect costs due to poor health outcomes and continuing presenteeism. Studies estimating healthcare costs [16, 56, 57] reported diabetes‐related costs to be 2.6 times higher than non‐diabetes conditions, excluding hidden self‐care costs [57]. Some technologies (e.g., CGM, insulin pumps) and newer glucose lowering drugs (e.g., SGLT‐2, GLP‐1) were considered cost‐effective with reduced acute hyper/hypoglycaemic episodes and hospitalisations [58, 59, 60, 61]. In Hong Kong, many patients have to pay out‐of‐pocket for these technologies and therapeutics due to insufficient insurance coverage [53]. Indeed, in many jurisdictions, diabetes is excluded from private insurance or covered with high premium. Our younger employees yearned for affordable insurance for better access to specialists, technologies and therapeutics. A few suggested conditional insurance schemes (e.g., linked to HbA_1c_ targets) as a potential model to reduce insurer risk while incentivising adherence. In the United States, uninsured adults with diabetes receive fewer recommended services and delayed care [62, 63]. Reducing financial strain may reduce cognitive bandwidth, enabling individuals to focus on proactive health behaviours. Whether these additional financial support would improve health outcomes and reduce presenteeism warrants further investigations.

### 4.4 Strengths and Limitations

Notable strengths include the involvement of a multi‐disciplinary expert panel (psychologist, health economist, public health specialist and clinicians) and the utilisation of methodological triangulation (integration of quantitative and qualitative data), which enhanced the credibility and validity of the findings. The high questionnaire completion rate (94%) supports the reliability of the survey data [10]. Qualitative analysis was conducted by trained researchers following Braun and Clarke's (2006) six‐step framework [29], with reporting aligned to the Consolidated Criteria for Reporting Qualitative Research (Table S6) to ensure methodological rigour. Participants exhibited diverse clinical characteristics, minimising selection bias and improving generalizability. Neither interviewer had clinical or personal ties to respondents and confidentiality reassurances promoted open discussion.

This study is not without limitations. Hospital‐based recruitment may over‐sample participants with complex disease than those from primary care. The qualitative subsample demonstrated higher distress and lower HRQoL than the broader YOD cohort. This may be attributable to maximum variation sampling that balanced participants by complications and insulin use, shifting the group averages in a generally low‐burden sample. As distress and perceived barriers were central outcomes, findings reflect more burdened individuals rather than being representative of the entire YOD population. Qualitative insights are hypothesis‐generating and intended to inform the development of systematic measurement approaches in future studies involving larger, representative populations. The single‐item WHO‐HPQ may be prone to social desirability bias. However, it remains a validated, widely used and pragmatic measure of presenteeism in clinical research [19]. The effect size between emotional burden and presenteeism was only modest, calling for careful interpretation. However, in a population with high prevalence of YOD and distress, the cumulative impact of emotional strain on productivity can become significant.

## 5 Conclusion

Among Chinese individuals with YOD, long working hours, inflexible workplace policies, entrenched presenteeism norms and insurance exclusions create a challenging context for optimal care. One in three participants experienced diabetes distress, which was independently associated with higher presenteeism. There are multiple personal, family, occupational and societal barriers for having access to optimal care. These were compounded by stigma, cultural expectations and suboptimal patient–provider communication. Participants proposed several mitigation strategies, including strengthening public awareness through early education, promoting earlier detection (e.g., through genetic testing where appropriate) and supporting informed decision‐making. Collectively, these findings underscore the need for a patient‐centred, multidimensional and multi‐stakeholder approach to address unmet needs and improve health and work‐related outcomes. The policy and intervention strategies suggested by participants warrant further evaluation in larger, representative samples.

## Funding

The study was supported by the Health and Medical Research Fund Commissioned Research Grant (CFS‐CUHK2).

## Ethics Statement

This study was approved by the Chinese University of Hong Kong—New Territories East Cluster Clinical Research Ethics Committee (reference number: 2019‐080‐T) and complied with the Declaration of Helsinki. Participants gave informed consent to participate in the study before taking part.

## Conflicts of Interest

J.C.N.C. reported receiving grants (through institutions) and/or honoraria for providing consultancy or giving lectures from Abbott, Applied Therapeutics, AstraZeneca, Bayer, Biomea, Boehringer Ingelheim, Eli Lilly, Hua Medicine, Merck, Novo Nordisk, Powder Pharmaceuticals, Roche, Sanofi, Servier and Zuellig Pharma Commercialisations. She is the Chief Executive Officer (pro bono) of the Asia Diabetes Foundation that developed the web‐based JADE platform for implementation of data‐driven diabetes management. She is a co‐inventor of patents owned by the Chinese University of Hong Kong with claims of using biomarkers and mathematical models to predict diabetes and its complications. J.C.N.C. and R.C.W.M. are co‐founders of GemVCare, a biotechnology company, with partial support from the Hong Kong Government, which uses biogenetic markers and information technology to implement precision diabetes care and prevention through partnerships. J.N.M.L. received research funding from Roche Diagnostics and Johnson and Johnson Ltd. for projects that are unrelated to the work presented in this manuscript. A.P.S.K. has received research grants and/or speaker honoraria from Abbott, Astra Zene ca, Bayer, Biomea, Boehringer Ingelheim, Dexcom, Eli‐Lilly, Kyowa Kirin, Merck Serono, Merck Sharp & Dohme, Nestle, Novo‐Nordisk, Pfizer, Sanofi and Zuellig Pharma.

## Supporting information


**Appendix S1.** Supporting Information methods.
**Appendix S2:** Interview guide: English (translated) focus group discussion (FGD) interview guide.
**Table S1:** Demographic characteristics of participants in the substudy of the PRISM‐RCT stratified by their employment status.
**Table S2:** Individual hierarchical regression models on the associations of presenteeism with patient reported outcomes measures (PROMs).
**Table S3:** Demographic characteristics of employed participants in the substudy of the PRISM‐RCT, stratified by their participation in the focus group discussion (FGD).
**Table S4:** False discovery rate (FDR) adjusted *p*‐values on the comparisons between high versus low presenteeism, unemployed versus employed and non‐FGD versus FGD participants.
**Table S5:** Example quotes from transcripts categorised by themes and subthemes of contributing factors and healthcare policies.
**Table S6:** Consolidated criteria for reporting qualitative studies (COREQ): 32‐item checklist.

## Data Availability

Due to legal restrictions, patient‐level data cannot be made publicly available. Aggregated data may be available upon reasonable request with research proposal. Requests to access the dataset should be directed to jchan@cuhk.edu.hk
