# Supplementary material for: Multi‐Dimensional Barriers for Work Productivity and Possible Solutions to Overcome Presenteeism in Young‐Onset Diabetes: A Mixed‐Methods Study
Source: Diabetes Obes Metab. 2026 Aug 26;28(11):10540–54. doi: 10.1111/dom.71158 (PMC13646383; doi:10.1111/dom.71158)
Supplement: Supplementary file 1 [file DOM-28-10540-s001.docx]

**Multi-dimensional Barriers for Work Productivity and Possible Solutions to Overcome Presenteeism in Young-Onset Diabetes: A Mixed-Methods Study**

**Supplementary Materials**

**Supplementary Materials**

**Table of Contents**

Supplementary appendix 1: Supplementary methods	3

1.1.	Overview of the Precision Medicine to Redefine Insulin Secretion and Monogenic Diabetes-Randomized Controlled Trial (PRISM-RCT) study	3

1.2.	Description of Braun and Clarke’s six-step framework	4

1.3.	Covariates being adjusted in multivariate analysis	4

1.4.	Roles, composition and reflexivity of research team	5

Supplementary appendix 2. Interview guide: English (translated) focus group discussion (FGD) interview guide	6

Table S1. Demographic characteristics of participants in the substudy of the PRISM-RCT stratified by their employment status	10

Table S2. Individual hierarchical regression models on the associations of presenteeism with patient reported outcomes measures (PROMs)	12

Table S3. Demographic characteristics of employed participants in the substudy of the PRISM-RCT, stratified by their participation in the focus group discussion (FGD)	14

Table S4. False Discovery Rate (FDR) adjusted p-values on the comparisons between high vs. low presenteeism, unemployed vs. employed, and non-FGD vs. FGD participants.	17

Table S5: Example quotes from transcripts categorized by themes and subthemes of contributing factors and healthcare policies	19

Table S6. Consolidated criteria for reporting qualitative studies (COREQ): 32-item checklist	24

# Supplementary appendix 1: Supplementary methods

## 1.1. Overview of the Precision Medicine to Redefine Insulin Secretion and Monogenic Diabetes-Randomized Controlled Trial (PRISM-RCT) study

The PRISM study is a three‑year pragmatic randomized controlled trial (RCT) that integrates the use of biogenetic markers (autoantibodies, fasting c-peptide, targeted sequencing for monogenic diabetes genes, and genome-wide genotyping) to derive polygenic risk scores, into algorithm-guided, specialist-led intensive treatment program for one year, followed by annual review over the subsequent two years, compared with usual care (1). The outcome measures included all diabetes-related complications [cardiovascular disease (coronary heart disease, heart failure, stroke, and peripheral artery disease), chronic kidney disease, albuminuria, retinopathy, visual impairment, sensory neuropathy, and all-cause death], attainment of multiple treatment targets and patient reported outcome measures (PROMs: Depression, Anxiety and Stress Scale, EQ-5D-3L, Chinese Diabetes Distress Scale, Summary of Diabetes Self-Care Activities, Perceived Personal Control, Patient Health Questionnaire-9). The patient recruitment commenced in 2020-2021 with year-3 follow-up.

*Summary of findings on psychological distress and self-management of the PRISM-RCT study*

Compared to the JADE-only (control) group, the JADE-PRISM (intervention) group showed greater improvements in the physical-related distress and regimen- and social-support-related distress at year 1 (2). Results of evaluation of PROMs can be found in the published PRISM-RCT paper (2).

In this sub-cohort of the PRISM-RCT who were actively employed (N=465), 206 (44%) and 259 (56%) individuals were randomised to JADE-only (control) and JADE-PRISM (intervention) groups respectively.

## 1.2. Description of Braun and Clarke’s six-step framework

| **Steps** | **Description** |
| --- | --- |
| 1. Familiarisation with data | Read and re-read the raw data (i.e., transcripts), understanding the in-depth and context, and create early insights. |
| 2. Generating initial codes | Systematically code the data to identify and summarize meaningful segments of text using software (e.g., NVivo) or manual coding. |
| 3. Searching for themes | Grouping the coded data into potential themes, with data patterns representing or relating to the research question. Tools such as mind maps or thematic maps may help in organising the themes. |
| 4. Reviewing themes | Refine the identified themes by reviewing them against the dataset to reassure internal coherence, clarity, and accuracy. This process may result in merging, splitting, or discarding themes. |
| 5. Defining and naming themes | Define the theme with details on its relationship with the research question. Naming of the themes should be concise and representative. Detailed description of the themes is necessary with supporting quotations to increase clarity. |
| 6. Writing the report | Writing up the findings through a coherent narrative with detailed description of the themes, data extraction and analytic commentary. Provide a comprehensive and insightful interpretation around the research question. |

## 1.3. Covariates being adjusted in multivariate analysis

| **Models** | **Covariates** |
| --- | --- |
| Model 1 | Demographic factors (age, sex, diabetes duration, education level, childcare responsibilities, group assignment: JADE-only vs JADE-PRISM) |
| Model 2 | Model 1 + cardiometabolic risk factors (body mass index, high-density lipoprotein cholesterol, low density lipoprotein cholesterol, systolic blood pressure, diastolic blood pressure, triglycerides, estimated glomerular filtration rate, urine albumin-to-creatinine ratio, HbA_1c_, and fasting plasma glucose) |
| Model 3 | Model 2 + medication use (oral glucose lowering drugs, insulin, glucagon-like-peptide-1 receptor agonists, blood pressure lowering drugs, renin–angiotensin system inhibitors, lipid‑lowering drugs, and statin). |

## 1.4. Roles, composition and reflexivity of research team

| **Researchers** | **Role** | **Professional background and reflexivity** |
| --- | --- | --- |
| Researcher 1  KTCW  (Female) | Data collection & Data Coding | Researcher holds a MSc in psychology and with extensive experience in qualitative research. She acknowledged that her academic training and personal perspectives could potentially influence data interpretation. Reflexivity was maintained through regular debriefing and discussing with researchers who had expertise in other fields to mitigate the influence of her perspectives on data interpretation. The researcher declared no prior professional and personal relationships with the participants. |
| Researcher 2  JNML  (Female) | Data collection & Data Coding | The researcher holds a PhD in Health Economics with extensive experience in qualitative and quantitative research. She acknowledged that her professional training and personal perspectives could influence data interpretation. Reflexivity was maintained through regular debriefing and discussion with researchers who had expertise in other fields to mitigate the influence of her perspectives on data interpretation. The researcher declared no prior professional and personal relationships with the participants. |
| Researcher 3  SCSC  (Male) | Data Coding | The researcher holds an MBChB degree and acknowledged that professional training and personal experiences could influence data interpretation. Reflexivity was maintained through regular team debriefings and interdisciplinary discussions to minimize potential bias. The researcher declared no prior clinical or personal relationships with the participants. |
| Researcher 4  NSW  (Female) | Data Coding |  |

# Supplementary appendix 2. Interview guide: English (translated) focus group discussion (FGD) interview guide

**Precision Medicine to Redefine Insulin Secretion and Monogenic Diabetes (PRISM) in Chinese Patients with Young Onset Diabetes**

**Economic impact of diabetes and related complications (EIDC) substudy**

Interview Guideline (English Version)

1. *Introduction*

- First of all, thank you for taking time and welcome to participate in the interview for “Precision Medicine to Redefine Insulin Secretion and Monogenic Diabetes – Economic Impact of Diabetes and Related Complications Substudy”. I am the researcher of this Project (*introduce interview’s name, background and experience*).
- It is estimated that approximately 5-7% of the working population in Hong Kong have diabetes, and the number is increasing. Therefore, we aim to estimate the economic impact of diabetes and its related complications (direct hospitalisation costs and indirect costs of productivity lost), and explore how people with diabetes cope with clinical conditions in the workplace.
- Through focus group discussions, we hope to explore risk factors and coping strategies for people with diabetes in the workplace. With regular monitoring of blood glucose levels, coupled with a healthy diet and exercises, diabetes-related complications (such as stroke, heart attack, amputation, and kidney failure) are highly preventable. Research indicates that with the most effective self-management of diabetes, the cumulative number of hospital days by age 75 years can be reduced by 30%.
- People with diabetes require persistent self-management care (e.g., healthy eating, regular exercise, blood glucose monitoring, medication adherence) to prevent future hospitalisations due to adverse events. Working employees with diabetes spend at least one-third of the time at work each day. In addition to handling daily tasks, they must manage health conditions caused by diabetes-related complications. Physical and psychosocial support provided by employers and colleagues at work is equally important.
- The findings of the present study will help policymakers plan for the resources allocation and develop early intervention policies to reduce future medical and economic burden of diabetes on society.
- Your comments are very important to this study, especially given that you are currently employed, as well as having lived experience in diabetes management; therefore, we specifically invited you to participate in this interview.
- Participation in this study is completely voluntary, and you can withdraw anytime or refuse to answer any questions. This interview will last approximately 60-90 minutes. All information provided will be kept confidential and used only for research purposes, and your personal information will not be disclosed. For the sake of confidentiality, we will not include your name in the interview transcript. To ensure accurate collection of your valuable opinions, the entire interview will be recorded. **(Recording will begin after obtaining the participants’ consent)**

2. *Building Trust*

- Before we begin, please turn off your mobile phone or put it on silent mode to avoid disrupting the discussion.
- Could you please briefly introduce yourself? Such as you name (how would you like me to call you), your occupation, and your work environment.
- This interview is not an examination, there are no standard answers and no right or wrong answers. We simply want to understand your perspectives and opinions, so please treat it like a casual conversation.
- Your opinions are very helpful to the research, so we hope you will actively express your views.
- Do you have any questions about this interview? If not, let’s begin today’s interview.

3. *Core Discussion*

| **Main Discussion 1: Decision-making processes underlying presenteeism** | |
| --- | --- |
| **Questions** | **Follow-up questions** |
| Please recall your memory to the last time you went to work while sick due to diabetes or its complications (including physical or mental illness) | - How did you decide to go to work (presenteeism) instead of taking sick leave (absenteeism)? - What factors did you consider when deciding to go to work? - Manpower - Shift work system - Pressure from colleagues and supervisors - Job type and rank issues |
| Does going to work while being sick makes a difference to self-care and management of diabetes compared to regular work? | - Please describe in detail what happened that day?   (from waking up, breakfast, going to work, lunch, to leaving work, dinner, and going to bed)   - Was your diet different from usual? - Was your exercise level different? - Were your self-care and diabetes management methods different at work?   *E.g., did you pay more attention to or need to spend more time on taking oral medication and insulin injection, resting time, or other diabetes-related issues than usual?*   - Do you feel that going to work while being sick had any downstream impacts on your health? If you had taken the day off while being sick, would the impact have been reduced? - What do you think are the top three things that diabetes most significantly affect your ability to work normally? |
| Have you ever been absent from work due to diabetes? | - How many days of leave did you take in total last time due to diabetes or its complications (non-hospitalization)? - How many days of leave did you take in total last time due to hospitalization or surgery for diabetes or its complications? - Did your colleagues or supervisors show any concern or support? - Compared to non-hospitalization, did your absence due to hospitalization or surgery affect your general work efficiency or work habits after returning to work? Please explain in detail. |
| Has diabetes affected your mental states? | - If diabetes is affecting your mental well-being, please describe in detail its daily impact. - How do you cope? - Did your family members, colleagues, or supervisors offer any care or support? - Did you improve your mood because of them? |
| **Main Discussion 2: Influence of workplace and human-resource policies on employees’ ability to manage their health** | |
| Have your colleagues and supervisors shown any care or support? | - Have your colleagues or supervisors noticed that you are coming to work or absent due to diabetes or its complications? - Have they shown any care or support for you? - Have they helped alleviate the work-related inconveniences caused by diabetes or its complications (e.g., jobs requiring physical labor or jobs that conflict with your self-care schedule)? |
| Does the company have policies that take into account the needs of people with diabetes? | - Do you think your company has policies aimed at alleviating the psychological and physical stress experienced by employees with diabetes? - What policies are in place or could be improved? - Does your company’s sick leave policy influence your decision to work while suffering from diabetes-related complications? Such as, sick leave application? Or insufficient sick leave available? - Does your work environment negatively impact your daily self-management of your diabetes? Are your employer aware of this? Have they made any adjustment? - Does your company provide health insurance? If so, - Does your company’s health insurance cover your diabetes-related medical expenses? - Does your company’s health insurance allow you to see a private or public doctor? - If your health insurance allows consultation with private doctors, what factors influence your decision to seek treatment at a public hospital? |
| **Main Discussion 3: Socioeconomic impact of diabetes and its complications on employees living with the condition** | |
| Does diabetes or its complications place a financial burden on you? | - How much out-of-pocket expenses (excluding health insurance) did you pay for diabetes-related medical expenses last year? - Have you been hospitalized or had surgery due to diabetes? Did your health insurance cover these expenses? - How much did you ultimately pay out-of-pocket for that hospitalization or surgery? Did it put a financial burden on you (how much of your monthly income go to out-of-pocket expenses for diabetes care)? - Did it affect your quality of life or daily meals that months? |
| The associated costs of follow-up visits or hospitalizations for diabetes or its complications (transportation, taking time off work for medical treatment)? | - Besides medical expenses, how much time or money do you spend on each doctor’s visit? - Transportation - Taking time off work for medical follow-up - Finding someone to take care of children or elderly relatives |
| Have you ever needed someone to care for you because of diabetes or its complications? (Including family members, caregivers, or domestic helpers) | - How long did you need to be cared for? Was it long-term or short-term? Please specify. - Who is caring for you? - If it is family, have they taken time off work to care for you? If so, approximately how many days on average? |
| Do you have children or elderly people to take care of? | - Has diabetes affected your ability to care for them? - Did diabetes or its complications affect your ability to take care of your family (dependents) in the past year? - How did you cope? - Did you ask for help? If so, how much time did your friends or family members spend helping you care for your dependents? - Did they take time off work to help? If so, for how many days? - If they did not come for help, what activities were they engaged in during that time? |
| Have you changed your daily habits or activities because of diabetes or its complications? | - Do you have things you used to do frequently before you were diagnosed with diabetes or its complications but are no longer able to do due to your condition? - Diet - Time spent on regular follow-up visits - Outdoor activities - Work-related activities - Social engagements with friends - Family gatherings - Mental health impact, reluctance to go out or move around - Others? - For the options mentioned above, how much would you be willing to pay each month to re-engage in these activities? |

4. *Summary/Suggestions*

- What do you consider to be the biggest socioeconomic impact of diabetes on you? How do you think healthcare, company policies, insurance policies, or government policies can alleviate these burdens?
- What improvements do you think you can make in your daily self-care or diabetes management to reduce the socioeconomic impact of diabetes on you? What policies can encourage you to make improvements in your daily self-care or diabetes management?

5. *Conclusion*

- Thank you very much for taking time to attend this interview.
- The audio recording of today’s discussion will be transcribed and then deleted. Your name will be kept completely confidential and will not appear in any reports.
- Do you have any questions about this research? If not, thank you again for your valuable time, comments, and assistance with this research.

# Table S1. Demographic characteristics of participants in the substudy of the PRISM-RCT stratified by their employment status.

|  | | **Frequency (%) / Mean±SD / Median (95% CI)** | | | | **p-value** |
| --- | --- | --- | --- | --- | --- | --- |
|  | | **N** | **Unemployed**  **(N=103)** | **N** | **Employed**  **(N=465)** |  |
| **Demographic characteristics** | |  |  |  |  |  |
| Age (years) | | 103 | 42.6±10.9 | 465 | 43.4±6.4 | 0.275 |
| Men (n, %) | | 103 | 41 (39.8) | 465 | 278 (59.8) | **<0.001** |
| College or above (n, %) | | 103 | 34 (33.0) | 465 | 182 (39.1) | 0.295 |
| Taking care of children < 18-year (n, %) | | 103 | 50 (48.5) | 465 | 155 (33.3) | **0.005** |
| Current or former tobacco use (n, %) | | 103 | 44 (42.7) | 465 | 153 (32.9) | 0.075 |
| Never drank alcohol (n, %) | | 103 | 70 (68.0) | 465 | 265 (57.0) | 0.053 |
| Family history of diabetes (n, %) | | 101 | 83 (82.2) | 458 | 367 (80.1) | 0.74 |
| **Metabolic risk factors** | |  |  |  |  |  |
| Age at diagnosis (years) | | 103 | 31.4±7.7 | 465 | 33.0±6.3 | **0.026** |
| Duration of diabetes (years) | | 103 | 10.8±6.0 | 465 | 10.9±6.0 | 0.847 |
| Fasting plasma glucose (mmol/L) | | 103 | 8.2±3.5 | 463 | 7.4±2.4 | **0.003** |
| HbA_1c_ (%) | | 102 | 7.5±1.7 | 463 | 7.3±1.5 | 0.096 |
|  | HbA_1c_ < 7% (n, %) | 102 | 47 (46.1) | 463 | 239 (51.6) | 0.366 |
| Body mass index (kg/m^2^) | | 103 | 28.0±5.4 | 462 | 28.0±5.3 | 0.898 |
| Waist circumference (men, cm) | | 35 | 105.6±12.3 | 216 | 99.8±12.1 | **0.009** |
| Waist circumference (women, cm) | | 49 | 93.9±13.2 | 152 | 93.7±12.8 | 0.921 |
| Systolic BP (mmHg) | | 103 | 122.6±16.3 | 462 | 125.4±15.0 | 0.093 |
| Diastolic BP (mmHg) | | 103 | 74.4±11.2 | 462 | 75.8±10.4 | 0.233 |
| LDL-cholesterol (mmol/L) | | 100 | 2.1±0.7 | 453 | 2.1±0.8 | 0.93 |
| Triglycerides (mmol/L) | | 103 | 1.5 (1.0, 2.4) | 463 | 1.3 (0.9, 2.0) | **0.02** |
| eGFR (mL/min/1.73m^2^) | | 103 | 106.6 (93.3, 114.8) | 462 | 104.7 (89.4, 111.3) | 0.055 |
| Albuminuria (n, %) | | 103 | 38 (36.9) | 465 | 159 (34.2) | 0.055 |
| **Diabetes self-management and hypoglycaemia** | | | | | | |
| Self-reported hypoglycaemia in last 3 months (n, %) | | 103 | 30 (29.1) | 465 | 114 (24.5) | 0.396 |
| Self-monitoring of blood glucose/week (n, %) | | 103 | 34 (33.0) | 465 | 153 (32.9) | 1.000 |
| Physical activity ≥ 5 times/week (n, %) | | 103 | 18 (21.4) | 465 | 90 (24.4) | 0.665 |
| Good adherence to balanced diet (n, %) | | 103 | 32 (31.1) | 465 | 102 (21.9) | 0.065 |
| **Medication** | |  |  |  |  |  |
| Oral glucose-lowering drugs (n, %) | | 103 | 98 (95.1) | 465 | 437 (94.0) | 0.822 |
| Insulin (n, %) | | 103 | 32 (31.1) | 465 | 128 (27.5) | 0.547 |
| GLP-1 RA (n, %) | | 102 | 28 (27.5) | 458 | 118 (25.8) | 0.821 |
| Blood pressure-lowering drugs (n, %) | | 103 | 78 (75.7) | 465 | 365 (78.5) | 0.63 |
| Renin angiotensin system inhibitor (n, %) | | 102 | 71 (69.6) | 455 | 327 (71.9) | 0.737 |
| Lipid-regulating drugs (n, %) | | 103 | 84 (81.6) | 465 | 385 (82.8) | 0.875 |
| Statin (n, %) | | 102 | 77 (75.5) | 454 | 364 (80.2) | 0.357 |
| **History of complications and comorbidities** | | | | | | |
| Cardiovascular disease (n, %) | | 103 | 9 (8.7) | 465 | 44 (9.5) | 0.967 |
|  | Coronary heart disease (n, %) | 103 | 8 (7.8) | 465 | 29 (6.2) | 0.727 |
|  | Stroke (n, %) | 103 | 3 (2.9) | 465 | 11 (2.4) | 1.000 |
|  | Peripheral vascular disease (n, %) | 103 | 1 (1.0) | 465 | 6 (1.3) | 1.000 |
|  | Congestive heart failure (n, %) | 103 | 3 (2.9) | 465 | 2 (0.4) | 0.063 |
| Chronic kidney disease (n, %) | | 103 | 5 (4.9) | 465 | 32 (6.9) | 0.594 |
| End-stage kidney disease (n, %) | | 103 | 0 (0.0) | 465 | 5 (1.1) | 0.635 |
| All-site cancer (n, %) | | 103 | 12 (11.7) | 465 | 16 (3.4) | **0.001** |
| **Patient-reported outcomes** | |  |  |  |  |  |
| EQ-5D-3L utility score | | 103 | 0.8±0.2 | 456 | 0.9±0.1 | **<0.001** |
| EQ-5D-3L VAS (0-100) | | 103 | 68.5±18.1 | 460 | 69.1±16.6 | 0.742 |
| DASS-21 | |  |  |  |  |  |
|  | Depression (0-21) | 102 | 2.5 (1.0, 8.0) | 459 | 2.0 (0.0, 5.0) | **0.002** |
|  | Anxiety (0-21) | 103 | 3.0 (2.0, 7.0) | 460 | 2.0 (1.0, 5.0) | **0.002** |
|  | Stress (0-21) | 103 | 5.0 (1.0, 9.0) | 460 | 4.0 (1.0, 8.0) | **0.034** |
| C-DDS-15 | |  |  |  |  |  |
|  | Emotional burden (6-36) | 102 | 17.3±6.3 | 458 | 16.0±6.4 | 0.07 |
|  | Regimen- and social-related distress (6-36) | 102 | 17.7±5.7 | 458 | 17.0±5.8 | 0.254 |
|  | Physician-related distress (3-18) | 103 | 5.7±2.4 | 457 | 5.7±2.7 | 0.963 |
|  | C-DDS-15 ≥ 45 | 102 | 35 (34.3) | 455 | 135 (29.7) | 0.423 |
| SDSCA-15 | |  |  |  |  |  |
|  | Diet (0-7) | 103 | 3.8 (3.0, 4.8) | 460 | 3.5 (2.8, 4.5) | 0.053 |
|  | Exercise (0-7) | 103 | 2.0 (1.0, 4.0) | 460 | 2.0 (1.0, 3.5) | 0.704 |
|  | Blood glucose testing (0-7) | 103 | 1.0 (0.0, 2.0) | 458 | 1.0 (0.0, 3.0) | 0.432 |
|  | Foot care (0-7) | 103 | 3.7 (2.7, 5.3) | 460 | 3.3 (2.3, 5.0) | 0.083 |
|  | Adherence to medical advice (0-7) | 49 | 4.7 (4.0, 5.7) | 180 | 4.7 (2.3, 6.3) | 0.424 |
| CQ-4 Total (0-4) | | 103 | 4.0 (3.0, 4.0) | 454 | 4.0 (3.0, 4.0) | 0.892 |

C-DDS-15, Chinese version of Diabetes Distress Scale – 15 items; CQ-4, Compliance Questionnaire – 4 items; DASS-21, Depression, Anxiety, and Stress Scales – 21 items; EQ-5D-3L, EuroQol 5 Dimensions 3 Level Version; VAS, Visual Analogue Scale; SDSCA-15, Summary of Diabetes Self-Care Activities – 15 items; P-values indicates the comparison between the employed and unemployed groups.

# Table S2. Individual hierarchical regression models on the associations of presenteeism with patient reported outcomes measures (PROMs).

|  |  | **Model-1** | | | | **Model-2** | | | | **Model-3** | | | |
| --- | --- | --- | --- | --- | --- | --- | --- | --- | --- | --- | --- | --- | --- |
|  |  | ***β*** | **SE** | **95% CI** | ***p-value*** | ***β*** | **SE** | **95% CI** | ***p-value*** | ***β*** | **SE** | **95% CI** | ***p-value*** |
| **DASS-21** | |  |  |  |  |  |  |  |  |  |  |  |  |
|  | Depression | -0.09 | 0.03 | -0.16, -0.03 | **0.007** | -0.09 | 0.04 | -0.16, -0.02 | **0.010** | -0.06 | 0.04 | -0.14, 0.01 | 0.085 |
|  | Anxiety | 0.00 | 0.03 | -0.06, 0.07 | 0.896 | 0.01 | 0.04 | -0.06, 0.08 | 0.808 | 0.01 | 0.04 | -0.06, 0.08 | 0.802 |
|  | Stress | 0.00 | 0.03 | -0.06, 0.06 | 0.973 | 0.00 | 0.03 | -0.06, 0.06 | 0.980 | -0.01 | 0.03 | -0.08, 0.05 | 0.681 |
| **C-DDS-15** | |  |  |  |  |  |  |  |  |  |  |  |  |
|  | Emotional burden | -0.07 | 0.02 | -0.10, -0.03 | **<0.001** | -0.07 | 0.02 | -0.11, -0.04 | **<0.001** | -0.08 | 0.02 | -0.12, -0.04 | **<0.001** |
|  | Regimen-and-social-related distress | 0.01 | 0.02 | -0.03, 0.05 | 0.546 | 0.01 | 0.02 | -0.02, 0.05 | 0.478 | 0.03 | 0.02 | -0.01, 0.07 | 0.150 |
|  | Physician-related distress | 0.03 | 0.03 | -0.03, 0.10 | 0.326 | 0.03 | 0.03 | -0.03, 0.10 | 0.334 | 0.03 | 0.03 | -0.03, 0.10 | 0.331 |
| **EQ-5D-3L utility score** | | 2.19 | 0.61 | 1.00, 3.38 | **<0.001** | 2.21 | 0.62 | 0.99, 3.42 | **<0.001** | 1.50 | 0.68 | 0.16, 2.85 | **0.028** |
| **SDSCA** | |  |  |  |  |  |  |  |  |  |  |  |  |
|  | Diet | 0.05 | 0.10 | -0.15, 0.25 | 0.638 | 0.10 | 0.11 | -0.13, 0.32 | 0.406 | 0.10 | 0.12 | -0.13, 0.33 | 0.376 |
|  | Exercises | 0.02 | 0.07 | -0.12, 0.17 | 0.760 | 0.00 | 0.08 | -0.16, 0.16 | 0.993 | -0.02 | 0.08 | -0.18, 0.13 | 0.769 |
|  | Blood glucose testing | -0.09 | 0.07 | -0.22, 0.05 | 0.204 | -0.09 | 0.07 | -0.23, 0.05 | 0.208 | -0.12 | 0.08 | -0.27, 0.03 | 0.105 |
|  | Foot care | 0.17 | 0.07 | 0.03, 0.31 | **0.021** | 0.13 | 0.08 | -0.03, 0.28 | 0.113 | 0.13 | 0.08 | -0.03, 0.29 | 0.106 |
|  | Medication adherence | 0.06 | 0.07 | -0.07, 0.19 | 0.340 | 0.04 | 0.07 | -0.10, 0.18 | 0.587 | 0.02 | 0.08 | -0.15, 0.18 | 0.837 |
| **CQ-4** | | 0.09 | 0.07 | -0.05, 0.22 | 0.205 | 0.11 | 0.08 | -0.03, 0.26 | 0.132 | 0.11 | 0.08 | -0.05, 0.26 | 0.171 |

Presenteeism, “*How would you rate your overall job performance on the days you worked during the past 4 weeks (28 days)*?” from World Health Organisation-Health and Work Performance Questionnaire (WHO-HPQ); DASS-21, Depression, Anxiety, and Stress Scales – 21 items; C-DDS-15, Chinese version of Diabetes Distress Scale – 15 items; EQ-5D-3L, EuroQol 5 Dimensions 3 Level Version; SDSCA-15, Summary of Diabetes Self-Care Activities – 15 items; CQ-4, Compliance Questionnaire – 4 items. Model-1: Demographics (age, sex, duration of diabetes, college or above, taking care of child, group assignment); Model-2: Model-1 + Cardiometabolic risk factors (BMI, HDL-C, LDL-C, SBP, DBP, TG, eGFR, HbA1c, fasting plasma glucose); Model-3: Model-2 + Medications (antidiabetic drugs, insulin, GLP-1, antihypertensive drugs, RASi, hyperlipidaemia agents, statin) were adjusted. P-values indicates the association between study variables and presenteeism. *Note.* Regressions were conducted by each PROMs (i.e., DASS-21, C-DDS-15, EQ-5D-3L utility score, SDSCA, and CQ-4) separately, with subscales of corresponding PROM entered to the model simultaneously.

# Table S3. Demographic characteristics of employed participants in the substudy of the PRISM-RCT, stratified by their participation in the focus group discussion (FGD).

|  | | | **Frequency (%) / Mean±SD / Median (95% CI)** | | | | **p-value** |
| --- | --- | --- | --- | --- | --- | --- | --- |
|  | | | **N** | **Non-FGD**  **(N=449)** | **N** | **FGD**  **(N=16)** |  |
| **Demographic characteristics** | | |  |  |  |  |  |
| Age (years) | | | 449 | 43.4±6.4 | 16 | 45.1±5.9 | 0.282 |
| Men (n, %) | | | 449 | 269 (59.9) | 16 | 9 (56.2) | 0.973 |
| College or above (n, %) | | | 449 | 170 (37.9) | 16 | 12 (75.0) | **0.006** |
| Taking care of children < 18-year (n, %) | | | 449 | 150 (33.4) | 16 | 5 (31.2) | 1.000 |
| Current or former tobacco use (n, %) | | | 449 | 150 (33.4) | 16 | 3 (18.8) | 0.339 |
| Never drank alcohol (n, %) | | | 449 | 256 (57.0) | 16 | 9 (56.2) | 1.000 |
| Family history of diabetes (n, %) | | | 442 | 356 (80.5) | 16 | 11 (68.8) | 0.400 |
| **Metabolic risk factors** | | |  |  |  |  |  |
| Age at diagnosis (years) | | | 449 | 33.0±6.3 | 16 | 33.6±6.8 | 0.739 |
| Duration of diabetes (years) | | | 449 | 10.9±6.0 | 16 | 12.1±7.1 | 0.427 |
| Fasting plasma glucose (mmol/L) | | | 448 | 7.4±2.4 | 15 | 7.2±1.8 | 0.754 |
| HbA_1c_ (%) | | | 448 | 7.3±1.5 | 15 | 7.2±1.1 | 0.864 |
|  | HbA_1c_ < 7% (n, %) | | 448 | 232 (51.8) | 15 | 7 (46.7) | 0.898 |
| Body mass index (kg/m^2^) | | | 446 | 28.1±5.3 | 16 | 26.1±3.5 | 0.131 |
| Waist circumference (men, cm) | | | 212 | 99.8±12.2 | 4 | 99.8±12.3 | 0.998 |
| Waist circumference (women, cm) | | | 145 | 93.8±12.9 | 7 | 91.6±12.1 | 0.652 |
| Systolic BP (mmHg) | | | 446 | 125.2±15.0 | 16 | 129.9±13.7 | 0.223 |
| Diastolic BP (mmHg) | | | 446 | 75.7±10.5 | 16 | 78.1±7.4 | 0.220 |
| LDL-cholesterol (mmol/L) | | | 439 | 2.1±0.8 | 14 | 2.1±0.8 | 0.930 |
| Triglycerides (mmol/L) | | | 448 | 1.3 (0.9, 2.0) | 15 | 1.4 (1.2, 2.6) | 0.118 |
| eGFR (mL/min/1.73m^2^) | | | 447 | 105.0 (90.3, 111.3) | 15 | 88.9 (77.5, 105.7) | **0.038** |
| Albuminuria (n, %) | | | 449 | 154 (34.3) | 16 | 5 (31.2) | 1.000 |
| **Diabetes self-management and hypoglycaemia** | | | | | | | |
| Self-reported hypoglycaemia in last 3 months (n, %) | | | 449 | 110 (24.5) | 16 | 4 (25.0) | 1.000 |
| Self-monitoring of blood glucose/week (n, %) | | | 449 | 149 (33.2) | 16 | 4 (25.0) | 0.679 |
| Physical activity ≥ 5 times/week (n, %) | | | 449 | 87 (24.3) | 16 | 3 (27.3) | 1.000 |
| Good adherence to balanced diet (n, %) | | | 449 | 97 (21.6) | 16 | 5 (31.2) | 0.543 |
| **Medication** | | |  |  |  |  |  |
| Oral glucose-lowering drugs (n, %) | | | 449 | 421 (93.8) | 16 | 16 (100.0) | 0.620 |
| Insulin (n, %) | | | 449 | 123 (27.4) | 16 | 5 (31.2) | 0.957 |
| GLP-1 RA (n, %) | | | 442 | 113 (25.6) | 16 | 5 (31.2) | 0.826 |
| Blood pressure-lowering drugs (n, %) | | | 449 | 353 (78.6) | 16 | 12 (75.0) | 0.971 |
| Renin angiotensin system inhibitor (n, %) | | | 439 | 315 (71.8) | 16 | 12 (75.0) | 1.000 |
| Lipid-regulating drugs (n, %) | | | 449 | 370 (82.4) | 16 | 15 (93.8) | 0.398 |
| Statin (n, %) | | | 438 | 349 (79.7) | 16 | 15 (93.8) | 0.286 |
| **History of complications and comorbidities** | | | | | | | |
| Cardiovascular disease (n, %) | | | 449 | 42 (9.4) | 16 | 2 (12.5) | 1.000 |
|  | Coronary heart disease (n, %) | | 449 | 27 (6.0) | 16 | 2 (12.5) | 0.597 |
|  | Stroke (n, %) | | 449 | 11 (2.4) | 16 | 0 (0.0) | 1.000 |
|  | Peripheral vascular disease (n, %) | | 449 | 6 (1.3) | 16 | 0 (0.0) | 1.000 |
|  | Congestive heart failure (n, %) | | 449 | 2 (0.4) | 16 | 0 (0.0) | 1.000 |
| Chronic kidney disease (n, %) | | | 449 | 30 (6.7) | 16 | 2 (12.5) | 0.688 |
| End-stage kidney disease (n, %) | | | 449 | 5 (1.1) | 16 | 0 (0.0) | 1.000 |
| All-site cancer (n, %) | | | 449 | 16 (3.6) | 16 | 0 (0.0) | 0.944 |
| **Patient-reported outcomes** | | |  |  |  |  |  |
| EQ-5D-3L utility score | | | 441 | 0.7±0.1 | 15 | 0.8±0.1 | **0.003** |
| EQ-5D-3L VAS (0-100) | | | 445 | 69.4±16.5 | 15 | 60.7±18.4 | 0.585 |
| DASS-21 | | |  |  |  |  |  |
|  | Depression (0-21) | | 444 | 2.0 (0.0, 5.0) | 15 | 2.0 (0.0, 7.0) | 0.639 |
|  | Anxiety (0-21) | | 445 | 2.0 (1.0, 5.0) | 15 | 3.0 (1.5, 7.0) | 0.285 |
|  | Stress (0-21) | | 445 | 4.0 (1.0, 8.0) | 15 | 7.0 (3.5, 11.0) | **0.041** |
| C-DDS-15 | | |  |  |  |  |  |
|  | Emotional burden (6-36) | | 443 | 16.0±6.4 | 15 | 17.4±9.0 | 0.402 |
|  | Regimen- and social-related distress (6-36) | | 443 | 16.9±5.7 | 15 | 19.3±6.5 | 0.108 |
|  | Physician-related distress (3-18) | | 442 | 5.7±2.6 | 15 | 7.7±4.3 | **0.003** |
|  | C-DDS-15 ≥ 45 | | 440 | 130 (29.5) | 15 | 5 (33.3) | 0.977 |
| SDSCA-15 | | |  |  |  |  |  |
|  | Diet (0-7) | | 445 | 3.5 (2.8, 4.5) | 15 | 4.5 (3.9, 5.4) | 0.055 |
|  | Exercise (0-7) | | 445 | 2.0 (1.0, 3.5) | 15 | 3.0 (1.5, 3.5) | 0.509 |
|  | Blood glucose testing (0-7) | | 443 | 1.0 (0.0, 3.0) | 15 | 1.0 (0.0, 2.8) | 0.962 |
|  | Foot care (0-7) | | 445 | 3.3 (2.3, 5.0) | 15 | 2.7 (2.5, 5.3) | 0.824 |
|  | Adherence to medical advice (0-7) | | 174 | 4.7 (2.3, 6.3) | 6 | 5.5 (3.9, 6.8) | 0.458 |
| CQ-4 Total (0-4) | | | 439 | 4.0 (3.0, 4.0) | 15 | 4.0 (3.0, 4.0) | 0.706 |
| **Work-related status** | | |  |  |  |  |  |
| Working schedule | | |  |  |  |  |  |
|  | | Shift work (n, %) | 449 | 80 (17.8) | 16 | 4 (25.0) | 0.687 |
|  | | Regular working hours (n, %) | 449 | 363 (80.8) | 16 | 12 (75.0) | 0.795 |
|  | | Self-employed (n, %) | 449 | 13 (2.9) | 16 | 0 (0.0) | 1.000 |
| Working hours per week (hours) | | | 446 | 48.9±14.0 | 16 | 47.4±14.2 | 0.674 |
| Job categories | | | 448 |  | 16 |  | 0.692 |
|  | | Executive, administrative, or senior manager |  | 33 (7.4) |  | 1 (6.2) |  |
|  | | Professional |  | 61 (13.6) |  | 4 (25.0) |  |
|  | | Technical support |  | 44 (9.8) |  | 3 (18.8) |  |
|  | | Sales |  | 34 (7.6) |  | 0 (0.0) |  |
|  | | Clerical and administrative support |  | 108 (24.1) |  | 4 (25.0) |  |
|  | | Service occupation |  | 88 (19.6) |  | 2 (12.5) |  |
|  | | Precision production and crafts worker |  | 26 (5.8) |  | 1 (6.2) |  |
|  | | Operator or labourer |  | 54 (12.1) |  | 1 (6.2) |  |
| Working environment | | |  |  |  |  |  |
|  | | Indoors sedentary work | 449 | 244 (54.3) | 16 | 10 (62.5) | 0.698 |
|  | | Indoors manual work | 449 | 137 (30.5) | 16 | 2 (12.5) | 0.465 |
|  | | Outdoors sedentary work | 449 | 37 (8.2) | 16 | 2 (12.5) | 0.885 |
|  | | Outdoors manual work | 449 | 62 (13.8) | 16 | 2 (12.5) | 1.000 |
| Sickness Attendance | | | 448 |  | 15 |  | 0.707 |
|  | | >5 times |  | 73 (16.3) |  | 3 (20.0) |  |
|  | | 2 to 5 times |  | 151 (33.7) |  | 5 (33.3) |  |
|  | | Once |  | 79 (17.6) |  | 1 (6.7) |  |
|  | | None |  | 145 (32.4) |  | 6 (40.0) |  |
| Presenteeism/Productivity loss (0-10) | | | 446 | 7.1±1.6 | 15 | 7.1±1.5 | 0.985 |
| Monthly salary (n, %) | | | 444 |  | 16 |  |  |
|  | | $0-$9,799 |  | 18 (4.1) |  | 1 (6.2) |  |
|  | | $9,800-$13,999 |  | 27 (6.1) |  | 0 (0.0) |  |
|  | | $14,000-$18,699 |  | 72 (16.2) |  | 1 (6.2) |  |
|  | | $18,700-$29,499 |  | 169 (38.1) |  | 8 (50.0) |  |
|  | | $29,500-$46,199 |  | 94 (21.2) |  | 4 (25.0) |  |
|  | | ≥$46,200 |  | 64 (14.4) |  | 2 (12.5) |  |

Presenteeism/Productivity loss, “*How would you rate your overall job performance on the days you worked during the past 4 weeks (28 days)*?” from World Health Organisation-Health and Work Performance Questionnaire (WHO-HPQ); C-DDS-15, Chinese version of Diabetes Distress Scale – 15 items; CQ-4, Compliance Questionnaire – 4 items; DASS-21, Depression, Anxiety, and Stress Scales – 21 items; EQ-5D-3L, EuroQol 5 Dimensions 3 Level Version; VAS, Visual Analogue Scale; SDSCA-15, Summary of Diabetes Self-Care Activities – 15 items; P-values indicates the comparison between the employed and unemployed groups.

# Table S4. False Discovery Rate (FDR) adjusted *p*-values on the comparisons between high vs. low presenteeism, unemployed vs. employed, and non-FGD vs. FGD participants.

|  | | **FDR adjusted p-value*** | | |
| --- | --- | --- | --- | --- |
|  | | **High vs. Low Presenteeism**  **(Table 1)** | **Unemployed vs. Employed**  **(Table S1)** | **Non-FDG vs. FDG**  **(Table S3)** |
| **Demographic characteristics** | |  |  |  |
| Age (years) | | 1.000 | 0.630 | 1.000 |
| Men (n, %) | | 0.671 | **0.009** | 1.000 |
| College or above (n, %) | | 1.000 | 0.649 | 0.141 |
| Taking care of children < 18-year (n, %) | | 1.000 | **0.041** | 1.000 |
| Current or former tobacco use (n, %) | | 1.000 | 0.230 | 1.000 |
| Never drank alcohol (n, %) | | 0.673 | 0.216 | 1.000 |
| Family history of diabetes (n, %) | | 1.000 | 0.971 | 1.000 |
| **Metabolic risk factors** | |  |  |  |
| Age at diagnosis (years) | | 1.000 | 0.144 | 1.000 |
| Duration of diabetes (years) | | 1.000 | 1.000 | 1.000 |
| Fasting plasma glucose (mmol/L) | | 1.000 | **0.029** | 1.000 |
| HbA_1c_ (%) | | 1.000 | 0.252 | 1.000 |
|  | HbA_1c_ < 7% (n, %) | 0.766 | 0.746 | 1.000 |
| Body mass index (kg/m^2^) | | 1.000 | 1.000 | 0.875 |
| Waist circumference (men, cm) | | 0.886 | 0.064 | 1.000 |
| Waist circumference (women, cm) | | 0.551 | 1.000 | 1.000 |
| Systolic BP (mmHg) | | 0.673 | 0.252 | 1.000 |
| Diastolic BP (mmHg) | | 0.886 | 0.581 | 1.000 |
| LDL-cholesterol (mmol/L) | | 1.000 | 1.000 | 1.000 |
| Triglycerides (mmol/L) | | 0.904 | 0.124 | 0.875 |
| eGFR (mL/min/1.73m^2^) | | 1.000 | 0.216 | 0.499 |
| Albuminuria (n, %) | | 1.000 | 0.971 | 1.000 |
| **Diabetes self-management and hypoglycaemia** | |  |  |  |
| Self-reported hypoglycaemia in last 3 months (n, %) | | 0.766 | 0.766 | 1.000 |
| Self-monitoring of blood glucose/week (n, %) | | 1.000 | 1.000 | 1.000 |
| Physical activity ≥ 5 times/week (n, %) | | 1.000 | 0.971 | 1.000 |
| Good adherence to balanced diet (n, %) | | 0.658 | 0.223 | 1.000 |
| **Medication** | |  |  |  |
| Oral glucose-lowering drugs (n, %) | | 1.000 | 1.000 | 1.000 |
| Insulin (n, %) | | 0.621 | 0.941 | 1.000 |
| GLP-1 RA (n, %) | | 1.000 | 1.000 | 1.000 |
| Blood pressure-lowering drugs (n, %) | | 1.000 | 0.971 | 1.000 |
| Renin angiotensin system inhibitors (n, %) | | 1.000 | 0.971 | 1.000 |
| Lipid-regulating drugs (n, %) | | 0.621 | 1.000 | 1.000 |
| Statin (n, %) | | 1.000 | 0.746 | 1.000 |
| **History of complications and comorbidities** | |  |  |  |
| Cardiovascular disease (n, %) | | 0.673 | 1.000 | 1.000 |
|  | Coronary heart disease (n, %) | 0.886 | 0.971 | 1.000 |
|  | Stroke (n, %) | 1.000 | 1.000 | 1.000 |
|  | Peripheral vascular disease (n, %) | 1.000 | 1.000 | 1.000 |
|  | Congestive heart failure (n, %) | 1.000 | 0.223 | 1.000 |
| Chronic kidney disease (n, %) | | 1.000 | 0.971 | 1.000 |
| End-stage kidney disease (n, %) | | 1.000 | 0.971 | 1.000 |
| All-site cancer (n, %) | | 1.000 | **0.018** | 1.000 |
| **Patient-reported outcomes** | |  |  |  |
| EQ-5D-3L utility score | | **0.016** | **0.009** | 0.114 |
| EQ-5D-3L VAS (0-100) | | 0.598 | 0.971 | 0.499 |
| DASS-21 | |  |  |  |
|  | Depression (0-21) | 0.416 | **0.018** | 1.000 |
|  | Anxiety (0-21) | 0.416 | **0.018** | 1.000 |
|  | Stress (0-21) | 0.312 | 0.170 | 0.499 |
| C-DDS-15 | |  |  |  |
|  | Emotional burden (6-36) | 0.551 | 0.225 | 1.000 |
|  | Regimen- and social-related distress (6-36) | 0.283 | 0.607 | 0.875 |
|  | Physician-related distress (3-18) | 0.886 | 1.000 | 0.114 |
|  | C-DDS-15 ≥ 45 | 0.886 | 0.766 | 1.000 |
| SDSCA-15 | |  |  |  |
|  | Diet (0-7) | 0.083 | 0.216 | 0.524 |
|  | Exercise (0-7) | 0.551 | 0.971 | 1.000 |
|  | Blood glucose testing (0-7) | 1.000 | 0.766 | 1.000 |
|  | Foot care (0-7) | 0.551 | 0.242 | 1.000 |
|  | Adherence to medical advice (0-7) | 0.971 | 0.766 | 1.000 |
| CQ-4 Total (0-4) | | 0.621 | 1.000 | 1.000 |
| **Work-related status** | |  |  |  |
| Working hours per week (hours) | | 0.283 | NA | 1.000 |
| Presenteeism/Productivity loss (0-10) | | **<0.001** | NA | 1.000 |

*False Discovery Rate (FDR) adjusted *p*-value

C-DDS-15, Chinese version of Diabetes Distress Scale – 15 items; CQ-4, Compliance Questionnaire – 4 items; DASS-21, Depression, Anxiety, and Stress Scales – 21 items; EQ-5D-3L, EuroQol 5 Dimensions 3 Level Version; VAS, Visual Analogue Scale; SDSCA-15, Summary of Diabetes Self-Care Activities – 15 items.

# Table S5: Example quotes from transcripts categorized by themes and subthemes of contributing factors and healthcare policies.

| **Contributing Factors** | |
| --- | --- |
| 1. **Personal factors** | |
|  | 1.1. ***Family collectivism*** |
|  | “*But for example, my partner, my wife, sometimes finishes work late, so I end up eating late, too. If it weren't for this discussion today, I would usually eat after 8:30pm... I mean, between 8:30pm and 9:00pm in the evening…but dinner gets pushed back later. This is one of the influences.*” – **respondent-14, FGD-4**  "*As… for taking medication, diabetes medication, I miss it about once a month on average. For the injection, since my partner and I supervise each other, I manage to keep up with them every day. It’s just that sometimes I might do it a bit later or a bit earlier.*" – **respondent-14, FGD-4**  "*Since I…after work, I have to take care of the kids, so I really don’t have much time to relax. This creates a vicious cycle that the less sleep I get, the harder it is to rest. This might be affecting my diabetes condition.*" – **respondent 10, FGD-2** |
|  | 1.2. ***Psychological and physical strain*** |
|  | "*When I'm tense or anxious, my blood sugar levels and other indicators tend to spike.*" – **respondent-16, FGD-4**  "*Sometimes when I lose control, I end up buying chips... Somehow, I think… eating certain chemical foods, like those with monosodium glutamate, actually makes me feel happier.*" – **respondent-15, FGD-4**  “*Actually, that’s true… even though I have read a bit about nutrition, but sometimes when I get home from work, I am really tired and just want to finish the meal quickly, showering quickly, then go for bed or rest.*” – **respondent-4, FGD-1**  “*…people in Hong Kong usually eat when they are under stress. The only way you can relieve your stress is through eating, and that’s how these illnesses arise. If you can’t regulate yourself…as it’s not a short-term issue…it will gradually lead to problems.*” **– respondent-11, FGD-3** |
| 2. **Working environment** | |
|  | 2.1. ***Concerns about the attitude of employers and colleagues*** |
|  | "*I, along with others, find that society generally accepts hypertension as a reason more than diabetes. Saying that you need to see a doctor for hypertension is less awkward in reality. It's like taking medicine; you don't want people to see it. Getting an injection is even more troublesome; you have to sneak off to the restroom to do it, or find a time before or after meetings. People will directly judge that your poor lifestyle habits are the reason for your illness, which leads to being overweight. They link poor lifestyle habits to diabetes, reinforcing the idea that if you have diabetes, it's because you can't control yourself or lack discipline. So, when people hear about diabetes, they think, 'Oh, that's bad; you must have done something wrong.' This negative perception naturally leads to a lower evaluation, and that's the reality.*" – **respondent-14, FGD-2**  “*Many years ago, people would actually care about this (having diabetes) because they felt that if you couldn't manage your own health, then in certain contexts — I've seen some articles saying that… you might be considered at a lower-level position. That means getting to a higher-level position, like promotion, might be affected by this.*” – **respondent-4, FGD-1**  “*I don't want people at work to see my weaknesses... I'm afraid that my boss will think, 'Oh, you're a bit weak' and you know how it goes — particularly those who are at older ages… always the first being pushed out…*” **respondent-7, FGD-2**  "*At some gatherings… when everyone is sharing food, the awkwardness comes. I honestly tell my colleagues, 'I’m sorry, I have diabetes, so I can't eat that much, or I need to cut out sugar.' In my view, I'm just telling my colleagues about my health condition; I am not being rude, I just genuinely can’t eat that much.*" – **respondent-6, FGD-2**  “*Since I am working in the field of mental health, when many members and… colleagues see you taking medicines, regardless what kind of medication it is, they actually accept it without saying anything.*” – **respondent-16, FGD-4**  “*Like I have a colleague who really doesn’t pay attention to his/her diet, eating lots of unhealthy foods. Other colleagues will be like ‘Hey, you better stop’, and even for beverages ‘You have run out of quota for this month’. They will remind me… or share some dietary information and tips*” – **respondent-4, FGD-1** |
|  | 2.2. ***Frequent work adjustment*** |
|  | "*How should I put it… You might see a nutritionist, meet with the diabetes nurses, and have follow-up appointments… which can be quite frequent. So, if you're taking so many days off, it's obviously not that appropriate.*" – **respondent-4, FGD-1**  "*The impact is…I've recently switched to a lighter job. My work used to be demanding, but now I've moved to a position with lower pay… but also a lower workload, means that it’s not as intense…I don’t need to worry about hypoglycaemic attack.”* – **respondent-7, FGD-2** |
|  | 2.3. ***Challenges in applying for leave*** |
|  | “*…the best is to have a medical cert, it feels relatively formal to prove you’re indeed having a disease, instead of pretending, as you are really (in need) seeing a doctor. And other colleagues might think ‘Oh, again? Taking time off to see a doctor, going for follow-up visits, so many things.’ I think having a medical cert is (better)…at least other colleagues will see it as a valid reason for absence.*” – **respondent-5, FGD-1** |
|  | 2.4. ***Work type and schedule*** |
|  | “*Because I’m also on shift, so sometimes I will get back home late. If you suggest I eat food with low-salt and low-sugar, the only thing I can do is try to control myself. I chose the dishes that aren’t too flavourful. And yes, I get told by my doctor every time I dine out.*” – **respondent-1, FGD-1**  “*I will be on shift, which means my shift might finish at around 9pm. The problem is that my mealtime will be irregular if I get home late at night. For example, if I work at 12:00pm, then I would have my dinner at 4:30pm. After finishing work at 9pm, I would need to eat again at around 10:00pm.*” – **respondent-15, FGD-4**  “*As for food, well, because I less likely to cook at home, I rarely bring my own lunch. I mostly order takeout and dine out in the evening before going home… and since I am on shift, sometimes I may get home late at night.*” – **respondent-1, FGD-1**  “*The most difficult part is lunch… it is hard to find a suitable lunch for people with diabetes when dining out.*” – **respondent-9, FGD-2** |
| 3. **Societal influences** | |
|  | 3.1. ***Relationships with care providers*** |
|  | “*Since I’ve used these (CGM)…and it’s been very effective. You can see your glucose tread in your daily life. After using this tool, I found that my glucose level isn’t peak at 2-hour, it’s actually peak at 1.5-hour… My glucose level was 5.3 mmol/L before eating, and if I ate something inappropriate, it went up to 9.x mmol/L at 1.5-hour and dropped to 7.5 mmol/L at 2-hour. But in fact, I went up to 9.x mmol/L which I didn’t realised I had eaten inappropriately.*” – **respondent-9, FGD-2**  “*But I actually found from my follow-up visits, not all the doctors know how to interpret the (CGM) reports. After explaining to them and said ‘oh, I’ve improved’, they still insist ‘no, you are not doing well, and you have to increase the dose’. I had to show them and explain again ‘no, look here, I’ve improved and you still want me to increase the dose?’, but then they said ‘ah, I don’t know how to read this’ and handed me a paper and ask me to write it (fingerprick glucose level) down again.*” – **respondent-4, FGD-1**  “*After seeing the nutritionist, you realise that they can’t really help you much because basically you know the thing, they provide basic information, mainly targeting the elderlies who had lower education level… like food pyramid that even primary school teaches. At the end, you are the one to control yourself. If you have to count every meal and every piece of meat, you won’t be happy. If you really want ice cream, you can choose to eat less rice or meat to balance, but doctors might not recommend. If you really want something, it’s ok to calculate the totals and adjust as needed. It’s not practical to count every piece of meat, that’s just infeasible. It’s not a recommendation if you cannot execute.*” – **respondent-14, FGD-4**  “*It’s (Doctors) different every time. We used to have follow-up visits every 3 months, perhaps my mum may not know how to tell the doctor her condition and the doctors didn’t review her medical history properly. He/She switched her to a new insulin, which caused hypoglycaemia, and almost led to hospitalisation… under life-threatening situation. Monitoring one’s glucose is very important, so it’s a key issue that having different doctors each visit creates more problems*” – **respondent-6, FGD-2** |
|  | 3.2. ***Medical costs*** |
|  | “*They recommend me to inject GLP-1, and this injection actually costs around… you can’t say it’s very expensive – it’s HKD$200 each, and I need once a week. I hesitated at first, but then I was thinking that my salary isn’t high, and I don’t want to make myself miserable.*” – **respondent-6, FGD-2**  “*For the CGM… like (brand), it costs over HKD$2000 a month just for glucose monitoring, and you even haven’t mentioned the medical expenses for the disease and the follow-up visits.*” – **respondent-14, FGD-4**  “*I was diagnosed with diabetes when I was 16, and they said they can’t provide it (insurance packages) to me because we ‘inherited diabetes’.”* – **respondent-5, FGD-1**  “*No insurance coverage…even if the agent tells you to give a try, which might have extra loadings, that extra loading is so significant that you can’t possibly afford it. Even for the Voluntary Health Insurance Scheme doesn’t help at all… because diabetes affects so many things and what can they actually cover?*” – **respondent-4, FGD-1** |
|  | 3.3. ***Societal perception*** |
|  | “*I really don’t…brave enough to tell others ‘ah, I was diagnosed with diabetes in my 30s’… even after being diagnosed for a few years, I only mentioned to close friends. I believe if I tell the elderlies that I had diabetes at my 30s, they will act like ‘huh? You have diabetes?’ and that upset me. They have the concept that young people shouldn’t be sick, you should be healthy and only be sick when you’re getting older. But their concept is not correct, and I mean some children can also be sick. They would be quite shocked if you told them someone has diabetes at their 30s, and so, I prefer not to share it.*” – **respondent-15, FGD-4.** |
| **Healthcare Policies** | |
| 1. **Prevention** | |
|  | 1.1. ***Educational programme*** |
|  | “*The earlier you start to control it, the better. However, I didn’t know what’s wrong and didn’t feel like anything was wrong as I don’t have family history. I used to exercise a lot and never think that I could have diabetes. So, I kept indulging. So as many students nowadays, you can only remind when they live indulgently, although no one listens. That’s the education problem.*” – **respondent-11, FGD-3**  “*It’s really being stigmatised… this (diabetes) is a disease similar to mental illness that is stigmatised. Isn’t it supposed to be normal? Everyone needs to take medications; you can get sick, and that’s a common thing. Why do people feel a need to hide and inject in the restroom?*” – **respondent-16, FGD-4**  “*I think prevention is very important. Sometimes… starting from the point that you even don’t know anything yet, primary or secondary school, we should introduce healthy eating habits. In fact, diabetes or gout often relates to your mouth, and having a healthy diet concept from a young age is essential*” – **respondent-3, FGD-1** |
|  | 1.2. ***Healthy lifestyle promotion*** |
|  | “*The government should encourage dietary therapy. They could encourage restaurants to offer options that’s tasty and also better for glucose. If you dine out now, there are basically no choices, everything available is high in sugar. You can’t avoid it but not everyone has the time to cook*” – **respondent-12, FGD-3** |
| 2. **Early detection for family members of patients with YOD** | |
|  | 2.1. ***Genetic screening and regular body check*** |
|  | “*Actually, I want the government to help my children because their risk is quite high. Is there anything that can be done to screen early, like testing for specific genes?”* – **respondent-13, FGD-3**  “*…or if they noticed certain genes, what we need to pay attention to, like doing more exercises? If we know, we’ll do; if we don’t know, we won’t.*” – **respondent-11, FGD-3** |
| 3. **Ongoing support** | |
|  | 3.1. ***Patient involvement in shared decision-making*** |
|  | “*I don’t know, if there are better medications, we don’t know unless the doctor tells us.*” – **respondent-11, FGD-3**  “*I think patients should be given a choice to try different medication. If one works well and even if it costs, say HKD$500/month, many people could probably afford it, right?*” – **respondent-12, FGD-3** |
|  | 3.2. ***Conditional insurance coverage*** |
|  | “*I know there are 1-2 insurance companies offer packages for chronic conditions, but the entry requirement and whether they approve depends on things like our blood glucose or our clinical reports. They may also add lots of loading fees… it’s still about age and health indicators. If you are saying support, I’d prefer buying with a certain claim amount instead of not allowing us entry at all. It’s quite unfair. If I can’t even get in, it feels like I’m already being kicked out and means that I can’t do anything.*” – **respondent-8, FGD-2** |
|  | 3.3. ***Peer support group*** |
|  | “*It feels lonely sometimes. If I can ask others, I realise that they are also having similar conditions, and that gives me some strength.*” – **respondent-7, FGD-2**  “*If we have group discussions like this… although it’s challenging to gather so many people, I think if there’s opportunity and time, it would be meaningful. It allows everyone to exchange ideas and share knowledge, like how to manage things or how to eat properly. However, my work schedule isn’t flexible, so I often miss calls or unable to reply messages. I may just respond later.*” – **respondent-9, FGD-2** |

# Table S6. Consolidated criteria for reporting qualitative studies (COREQ): 32-item checklist.

| **Item No** | **Guide Questions/Description** | **Reported on Page #** |  |  |
| --- | --- | --- | --- | --- |
| **Domain 1: Research team and reflexivity** | | |  |  |
| **Personal Characteristics** | | |  |  |
| 1. Interviewer/ facilitator | Which author/s conducted the interview or focus group? | p.5; Supplementary Appendix 1 |  |  |
| 2. Credentials | What were the researcher’s credentials? E.g., PhD, MD | Supplementary Appendix 1 |  |  |
| 3. Occupation | What was their occupation at the time of the study? |  |  |  |
| 4. Gender | Was the researcher male or female? |  |  |  |
| 5. Experience and training | What experience or training did the researcher have? |  |  |  |
| **Relationship with participants** | | |  |  |
| 6. Relationship established | Was a relationship established prior to study commencement? | Supplementary Appendix 1; p.14 |  |  |
| 7. Participant knowledge of the interviewer | What did the participants know about the researcher? e.g. personal goals, reasons for doing the research? |  |  |  |
| 8. Interviewer characteristics | What characteristics were reported about the interviewer/facilitator? e.g. Bias, assumptions, reasons and interests in the research topic |  |  |  |
| **Domain 2: study design** | | |  |  |
| **Theoretical framework** | | |  |  |
| 9. Methodological orientation and Theory | What methodological orientation was stated to underpin the study? e.g. grounded theory, discourse analysis, ethnography, phenomenology, content analysis | p.4-5 |  |  |
| **Participant selection** | | |  |  |
| 10. Sampling | How were participants selected? e.g., purposive, convenience, consecutive, snowball | p.4 |  |  |
| 11. Method of approach | How were participants approached? e.g., face-to-face, telephone, mail, email | p.5 |  |  |
| 12. Sample size | How many participants were in the study? | p.6 |  |  |
| 13. Non-participation Setting | How many people refused to participate or dropped out? Reasons? | p.6 (reason is not applicable) |  |  |
| 14. Setting of data collection | Where was the data collected? e.g., home, clinic, workplace | p.4-5 |  |  |
| 15. Presence of nonparticipants | Was anyone else present besides the participants and researchers? | N/A |  |  |
| 16. Description of sample | What are the important characteristics of the sample? e.g. demographic data, date | Table S3 |  |  |
| **Data collection** | | |  |  |
| 17. Interview guide | Were questions, prompts, and guides provided by the authors? Was it pilot tested? | Supplementary Appendix 2 |  |  |
| 18. Repeat interviews | Were repeat interviews carried out? If yes, how many? | N/A |  |  |
| 19. Audio/visual recording | Did the research use audio or visual recording to collect the data? | Audio. |  |  |
| 20. Field notes | Were field notes made during and/or after the interview or focus group? | p.5; Field notes made during interview and Chinese verbatim after interview. |  |  |
| 21. Duration | What was the duration of the interviews or focus group? | p.5 |  |  |
| 22. Data saturation | Was data saturation discussed? | p.5 |  |  |
| 23. Transcripts returned | Were transcripts returned to participants for comment and/or correction? | N/A |  |  |
| **Domain 3: analysis and findings** | | |  |  |
| **Data analysis** | | |  |  |
| 24. Number of data coders | How many data coders coded the data? | p.5; Supplementary Appendix 1 |  |  |
| 25. Description of the coding tree | Did the authors provide a description of the coding tree? | Figure 2 |  |  |
| 26. Derivation of themes | Were themes identified in advance or derived from the data? | p.5 |  |  |
| 27. Software | What software, if applicable, was used to manage the data? | p.5 |  |  |
| 28. Participant checking | Did participants provide feedback on the findings? | N/A |  |  |
| **Reporting** | | |  |  |
| 29. Quotations presented | Were participant quotations presented to illustrate the themes/findings? Was each quotation identified? e.g., participant number | Table S5 |  |  |
| 30. Data and findings consistent | Was there consistency between the data presented and the findings? | p.6-10 |  |  |
| 31. Clarity of major themes | Were major themes clearly presented in the findings? | p.6-10 |  |  |
| 32. Clarity of minor themes | Is there a description of diverse cases or a discussion of minor themes? | p.6-10 |  |  |

**References**

1.	O CK, Fan YN, Fan B, Lim C, Lau ESH, Tsoi STF, et al. Precision Medicine to Redefine Insulin Secretion and Monogenic Diabetes-Randomized Controlled Trial (PRISM-RCT) in Chinese patients with young-onset diabetes: design, methods and baseline characteristics. BMJ Open Diabetes Res Care. 2024;12(3).

2.	Luk AOY, O CK, Fan B, Lau ESH, Fan Y, Lim C, et al. Precision Medicine to Redefine Insulin Secretion and Monogenic Diabetes (PRISM): A Randomized Controlled Trial in Chinese Patients With Young-Onset Diabetes. Diabetes Care. 2026.
